# Embryo-scale tissue mechanics during *Drosophila* gastrulation movements

**DOI:** 10.1038/ncomms9677

**Published:** 2015-10-26

**Authors:** Matteo Rauzi, Uros Krzic, Timothy E. Saunders, Matej Krajnc, Primož Ziherl, Lars Hufnagel, Maria Leptin

**Affiliations:** 1 European Molecular Biology Laboratory Heidelberg, Meyerhofstrasse 1, 69117 Heidelberg, Germany; 2 Jožef Stefan Institute, Jamova 39, SI-1000 Ljubljana, Slovenia; 3 Faculty of Mathematics and Physics, University of Ljubljana, Jadranska 19, SI-1000 Ljubljana, Slovenia; 4 Erwin Schrödinger International Institute for Mathematical Physics, University of Vienna, Boltzmanngasse 9, A-1090 Vienna, Austria

## Abstract

Morphogenesis of an organism requires the development of its parts to be coordinated in time and space. While past studies concentrated on defined cell populations, a synthetic view of the coordination of these events in a whole organism is needed for a full understanding. *Drosophila* gastrulation begins with the embryo forming a ventral furrow, which is eventually internalized. It is not understood how the rest of the embryo participates in this process. Here we use multiview selective plane illumination microscopy coupled with infrared laser manipulation and mutant analysis to dissect embryo-scale cell interactions during early gastrulation. Lateral cells have a denser medial–apical actomyosin network and shift ventrally as a compact cohort, whereas dorsal cells become stretched. We show that the behaviour of these cells affects furrow internalization. A computational model predicts different mechanical properties associated with tissue behaviour: lateral cells are stiff, whereas dorsal cells are soft. Experimental analysis confirms these properties *in vivo*.

## Abstract (web summary)

It is unclear how cell movements coordinate ventral furrow formation at the start of gastrulation in flies. Here, using multiview light-sheet microscopy and cell immobilization, Rauzi *et al.* observe differential epithelial cell movements, which contribute to the dynamics and timing of mesoderm internalization.

## Graphical abstract

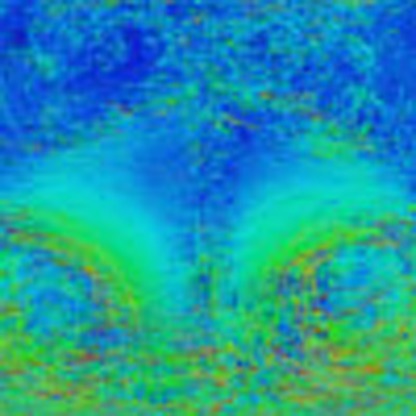

Reductionist approaches in developmental biology have been successful in elucidating the morphogenesis of organs and other cell assemblies and revealing the underlying genetic and molecular mechanisms of processes such as cell migration, epithelial folding and cell rearrangements. Such analyses have concentrated mainly on the activities inherent to the physiological unit being studied. Little is known on how the behaviours of the units are integrated with those of neighbouring cell populations or the whole organism. While it is intuitively clear that different parts of the body cannot operate in isolation, few studies^1^ have addressed the question how all tissues and cells in an organism cooperate to generate a properly structured animal.

Early gastrulation in *Drosophila* is particularly well suited for such an analysis, as its fundamental processes have been intensively studied and the underlying transcriptional programmes, which determine the behaviours of the participating cell populations, are well understood. The beginning of gastrulation is also very simple, involving a homogeneous epithelium in which the cells do not divide, delaminate or migrate. The epithelial structure is retained throughout a period of extensive tissue displacements.

The invagination of the mesoderm is controlled by a transcriptional programme that leads to the creation of a contractile actomyosin network at the apical–medial cortex that changes the shapes of a central band of mesodermal cells^2^,^3^,^4^,^5^,^6^,^7^,^8^,^9^,^10^,^11^,^12^. Genetic evidence supports the notion that the force for generating the furrow is created cell autonomously in the mesoderm^12^,^13^, although theoretical models have pointed to the difficulty in explaining the full internalization exclusively by apical constriction and have postulated contributions from cells outside the furrow^14^,^15^. We show that whole-body coordination of the mechanics of tissue behaviour is necessary for mesoderm invagination during *Drosophila* gastrulation. The behaviour and shape changes of cells belonging to different tissues correlate with their apical actomyosin architecture and with biomechanical tension predicted computationally and tested experimentally.

## Results

### Correlation of movements of cell groups during gastrulation

**Figure 1:**
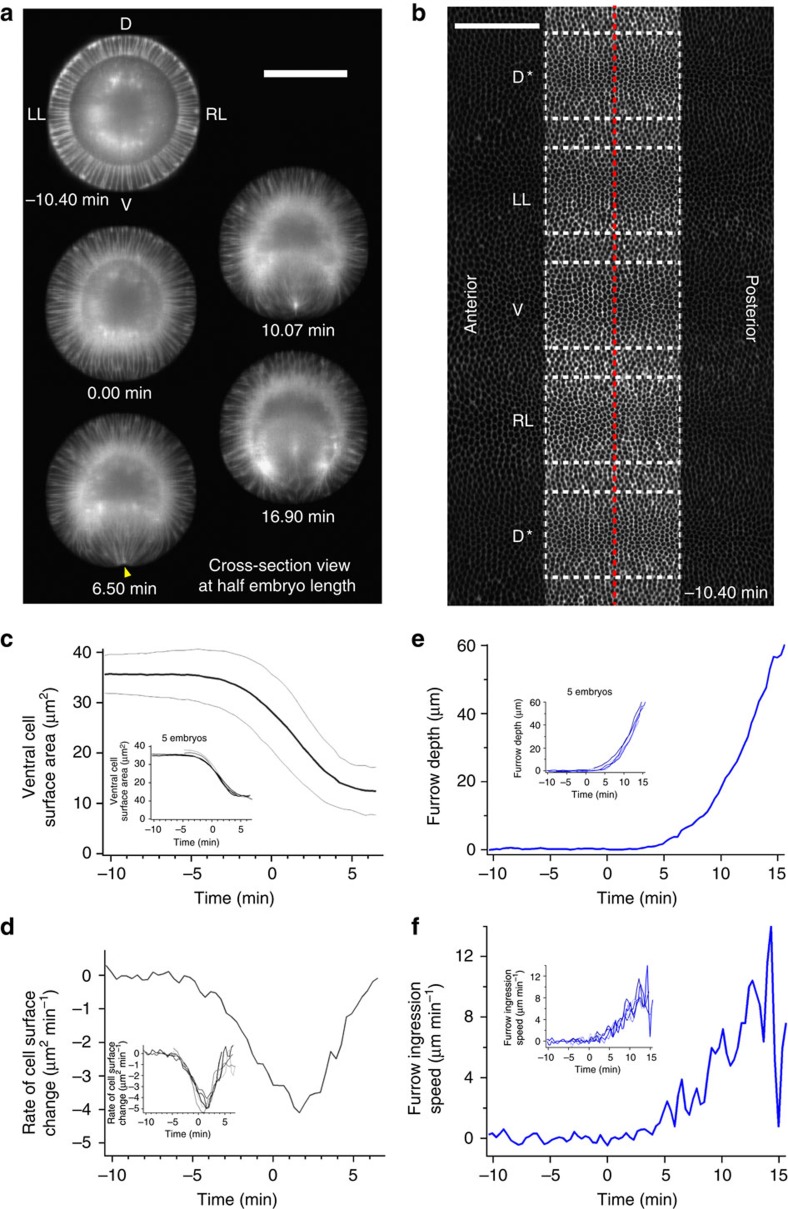
Embryo-scale views of the early stages of gastrulation. (**a**,**b**) Extractions of images from a 3D time-lapse reconstruction of a *Drosophila* embryo during early development. Cell outlines were imaged using Gap43::mCherry. (**a**) Embryo cross-section views from the 3D time-lapse reconstruction at five time points during mesoderm internalization. Dorsal, ventral, lateral left and lateral right cell groups are indicated as D, V, LL and RL, respectively. *t*=0 min corresponds to 20% of ventral cell apical constriction (Supplementary Fig. 3). Yellow arrowhead: initial indentation of the ventral furrow. (**b**) Cylindrical projection of the central region of an embryo. Dashed rectangles indicate the four areas used for quantitative analysis. The projection covers the azimuth range of 450° so that the dorsal side is shown twice (marked by an asterisk). The red line marks the position at which the kymograph was recorded. (**c**) Average and s.d. of ventral cell surface areas over time. (**d**) Rate of cell surface change over time. (**e**) Furrow depth over time. (**f**) Speed of furrow ingression over time. The insets in **c**–**f** show the results for five embryos. Scale bars, 100 μm.

To correlate cell shape and movement throughout the embryo requires high-spatial-resolution imaging of cell outlines with sufficient time resolution to track cells. Using multiview selective plane illumination microscopy (MuVi-SPIM)^16^, we obtain high-resolution three-dimensional (3D) images of embryos expressing the membrane marker Gap43::mCherry with a time resolution of ≈20 s per data set from late cellularization until complete mesoderm internalization (Fig. 1a). By the end of this period, the posterior midgut (PMG) begins to be displaced dorsally, but ectodermal cell intercalation has not yet started. We concentrate on the cell outlines at the apical side of cells, as this is where adherens junctions are located and forces responsible for tissue morphogenesis are generated^2^,^5^,^6^,^7^,^16^,^17^,^18^,^19^,^20^,^21^,^22^. For this purpose, we created a cartographic cylindrical view of the surface of the embryo (see Fig. 1b, Supplementary Figs 1 and 2, Supplementary Movies 1 and 2, and Methods section)^23^. Supplementary Figure 3b describes the timing framework we use for comparison between embryos. We set as *t*=0 min the time when a wild-type embryo has constricted 20% of the furrow-forming cells. We defined four regions for quantitative evaluation: ventral, left lateral, right lateral and dorsal (Fig. 1b), and focused on the central, 180-μm-wide region along the anterior–posterior (AP) axis of the embryo.

As previously shown^10^,^11^,^12^, an ∼10-cell-wide band of cells on the ventral side constrict apically (Fig. 1c and Supplementary Movie 3). The rate of constriction reaches a maximum when the cell surface is reduced on average by ≈40% (*t*≈2 min) and then decreases (Fig. 1d). To follow mesodermal cell movement beyond this point, we tracked the apex of the invaginating furrow on a cross-section view of the embryo (Supplementary Movie 4) and measured the furrow depth over time (Fig. 1e). The apex of the furrow moves towards the interior and the speed of internalization increases at ≈6 min (Fig. 1f). Thus, the maximum rates of apical constriction and furrow internalization are distinct in time, indicating that, in line with predictions from computational models (reviewed in refs 24, 25), apical constriction alone cannot account for furrow internalization.

**Figure 2:**
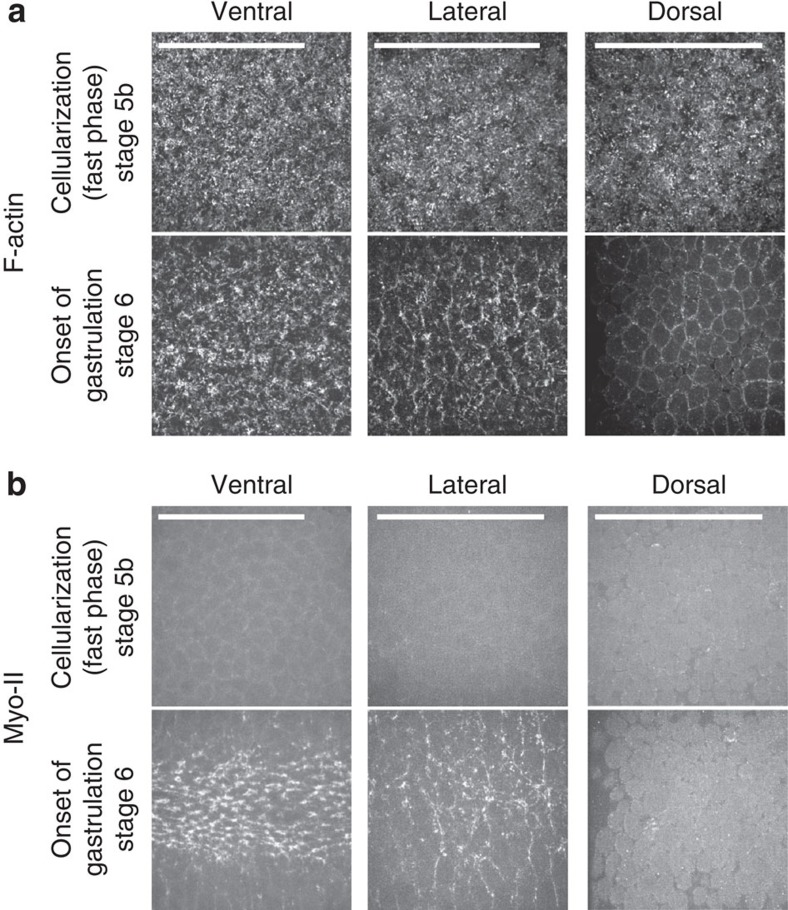
Differential distribution of the apical–medial actomyosin meshwork in the different cell populations. (**a**) F-actin, labelled with MoesinABD::GFP. (**b**) Myo-II, labelled with sqh::GFP in the ventral, lateral and dorsal regions at a stage just before gastrulation (stage 5b) and during gastrulation (stage 6). Scale bars, 50 μm.

**Figure 3:**
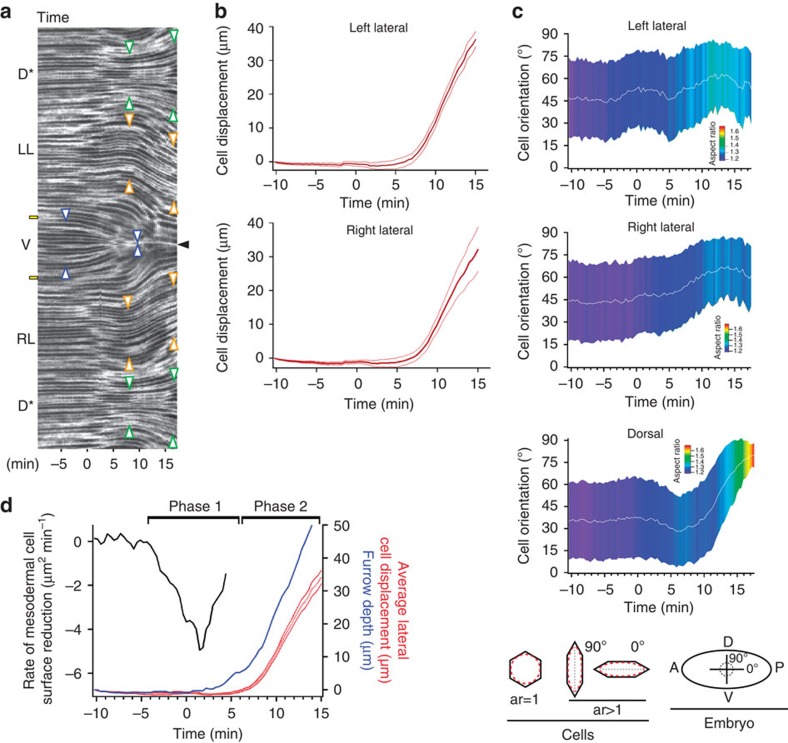
Differential behaviour of cell populations. (**a**) Kymograph taken at the centre of the embryo (Fig. 1b, red line). Arrowheads mark the edges of three cell populations. Blue: region with apical constrictions; orange: ventrally displacing lateral cells; and green: dorsal spreading cells. Black arrowhead: furrow midline where the two lateral tissues meet. Yellow rectangles: edge of the future mesoderm, determined as described in Supplementary Fig. 5b. The ‘*' stresses the fact that the same dorsal tissue is represented twice. (**b**) Average and s.d. of left lateral (LL) and right lateral (RL) cell displacement along the DV axis over time. Positive values indicate displacement towards the ventral side. (**c**) Average and s.d. of major axis orientation for the LL, RL and dorsal (D) cells. The diagram below the three panels illustrates the measurements that were made: the aspect ratio of each cell (left; colour in the panels indicates cell eccentricity, with red for the highest aspect ratios) and their orientation relative to the AP axis of the embryo (right). (**d**) Rate of change in apical cell surface area of ventral cells (black), depth of the furrow (blue) and displacement of the two lateral cell populations (red). The solid red lines show the average displacement along the DV axis of the RL and the LL cells in time. The dashed red line shows the average displacement of RL and LL. All quantifications of cell behaviour are based on measurements the of segmented 2D surface view of the embryo.

To keep the embryo intact during mesoderm invagination, the epithelium outside the furrow must respond by cell movements or cell shape changes. Different scenarios can be envisioned. At one extreme, the epithelium could act as a homogeneous cell sheet that is deformed by the movements of the mesoderm; alternatively, specialized subpopulations of cells might be mechanically different and respond or even actively contribute in a cell-type specific fashion. While the epithelium that makes up the early, pre-gastrulation embryo is homogeneous in its cell biological properties, differences in the distribution of the force-generating and shape-determining elements of the cells, namely, the actin cytoskeleton and the adherens junctions become detectable as gastrulation begins. The junctions in ventral cells are shifted from a subapical to an apical position^6^. Immediately afterwards, a medial actomyosin meshwork assembles at the apical cortex of the mid-ventral cells^2^,^7^. Comparing F-actin with Myo-II in ventral, lateral and dorsal cells, we find that before gastrulation (stage 5b), F-actin is uniformly distributed (Fig. 2a). At the onset of gastrulation (stage 6), medial–apical F-actin is strongly reduced in dorsal cells and partially reduced in lateral cells. Myo-II clusters are seen during stage 6 in ventral and lateral cells^2^,^16^, but not in dorsal cells (Fig. 2b). In the ventral cells, Myo-II clusters begin to appear and E-cadherin is relocated apically before the onset of apical constriction^6^,^8^; similarly, in lateral cells, these events occur just before the cells begin to displace ventrally (Supplementary Fig. 4a and Supplementary Movie 5). This shows that the subpopulations of cells along the dorso–ventral (DV) axis differ in their cytoskeletal and contractile scaffolds, and hence likely in their mechanical properties. To test whether these differences were reflected in differential cell behaviour in response to mesoderm invagination, we measured the displacement and shapes of the lateral and dorsal cells. Lateral and dorsal cells all retain their surface areas (Supplementary Fig. 4b). The two lateral populations are displaced towards the ventral midline over a distance of 35 μm (Fig. 3a,b), whereas the cells within each sheet maintain their positions relative to each other (Supplementary Fig. 4c and Supplementary Movie 6), and their aspect ratios remain largely unchanged (Fig. 3c). Thus, they are displaced as a coherent cohort of cells. In contrast, dorsal cells increase their aspect ratio and orient their long axes along the circumference (90°; Fig. 3a,d).

The fact that the lateral cells remain stationary during the initial formation of the furrow means that the cells between them and the constricting cells have to accommodate all of the surface reduction of the constricting cells. This population is part of the mesoderm, the peripheral, non-constricting mesodermal cells. As previously observed, and as evident in the movies (Supplementary Fig. 5), they indeed undergo a shape change by elongating towards the central mesodermal cells^10^,^12^,^26^,^27^.

In summary, we can subdivide the process of mesoderm invagination into two phases. During phase 1 (between −5 and +6 min), the central mesodermal cells constrict apically while the neighbouring, peripheral mesodermal cells elongate. In phase 2 (from 6±2 min onwards), the ventral furrow starts its rapid internalization, whereas the lateral cells move in a compact cohort towards the ventral midline and dorsal cells stretch along the DV axis (Fig. 3c,d). These results show that the blastoderm epithelium does not respond homogeneously to the invagination of the ventral furrow, supporting the notion that different cell populations have different properties. They also show that the movement of the lateral and dorsal cells does not contribute to the initial formation of the furrow (phase 1), confirming genetic results showing that the formation of the furrow does not depend on the activity of the ectoderm^12^,^13^.

### Effects of cell fate changes on gastrulation

Having seen that cells along the DV axis of the embryo behave differently during early gastrulation, we wanted to test the roles of these different behaviours for the proper progression of gastrulation. We took advantage of mutants to change the fates and therefore the properties of cell populations in the embryo. We first analysed embryos from mothers carrying the dominant *Toll* allele, *Toll*^*10B*^, in which all cells along the DV axis have the mesodermal fate. We used embryos from mothers that also carry a wild-type allele of the gene (*Toll*/*Toll*^*10B*^ heterozygotes), which imposes a slight asymmetry along the DV axis^12^,^28^. Such embryos have been shown to be able to make a ventral furrow, leading to the conclusion that dorsal and lateral cell fates are not essential for furrow formation^12^. This affords us an opportunity to test whether cells located in lateral and dorsal positions in the embryo, but that have a ‘wrong' (ventral) fate, respond to furrow formation in the same or a different manner compared with cells with proper lateral and dorsal fates.

In embryos from *Toll*/*Toll*^*10B*^ mothers, all cells are enriched apically in Myo-II and constrict apically (Supplementary Fig. 6a–d). The polarity resulting from the wild-type *Toll* allele leads to a broadband of cells on the ventral side constricting (Supplementary Fig. 6e). The cells on the lateral side are displaced ventrally, but this movement is reduced and different in character from that of the ectoderm in wild-type embryos (Supplementary Fig. 6f and Fig. 3a). The cells do not move as a compact cohort, but stretch along the DV axis. The dorsally located cells also stretch, but less than the lateral cells (Supplementary Fig. 6g,h). Thus, in this situation, the entire epithelium outside the constricting cells behaves as a more homogenous, deformable sheet, which is in clear contrast to the wild type. Therefore, the cohort-like behaviour of the lateral cells in the wild-type embryo must be determined by their cell fate rather than exclusively by the geometry of the embryo or their distance from the constricting ventral cells.

A number of other morphogenetic events occur in the embryo during the invagination of the mesoderm that might affect the overall coordination of tissue movement. A transverse fold, the cephalic fold, is formed behind the head region, and, at the posterior end, the invaginating PMG primordium translocates towards the dorsal side of the embryo (Supplementary Fig. 3a). To assess the effect of these processes, we analysed embryos in which they were abolished (embryos derived from mothers homozygous for the genes *bicoid, nanos* and *torso-like*, ‘*bnt'*). The cell fates along the DV axis are not affected and these mutants are known to internalize their mesoderm^29^ (Supplementary Movie 7). The differential cell behaviour along the DV axis is retained: the ectoderm moves as a cohort and the dorsal cells widen (Supplementary Fig. 7a–e). This shows that cephalic furrow formation and midgut movement are not necessary for these behaviours *per se*. Phase 1 of gastrulation occurs normally (Supplementary Fig. 7a,e,f). Phase 2 is also completed but our analysis uncovered an unexpected effect on timing: the internalization of the mesoderm and the displacement of the ectoderm are both delayed (Supplementary Fig. 7f,g and Supplementary Movie 7).

The finding that lateral cell displacement and mesoderm internalization are delayed even though apical constriction occurs normally shows that apical constriction alone is not sufficient to trigger these events on time. It suggests that the activity of the cephalic fold and the PMG contribute in determining the timing of lateral cell movement in the wild-type embryo. The fact that, as in the wild-type, lateral cell displacement and mesoderm internalization are correlated in time suggests that they are either causally related or both controlled by the same process. Finally, the lateral cells continue to move ventrally even after the furrow is completely internalized (Supplementary Fig. 7f,g) and in the absence of cell intercalation (which is abolished in *bnt* mutants^30^). This may mean either that lateral cell displacement is driven in part by mechanisms that are independent of mesoderm invagination, or that elastic stresses stored during mesoderm constriction can persist and contribute to driving ectodermal movement.

### Interaction between lateral and ventral tissue

**Figure 4:**
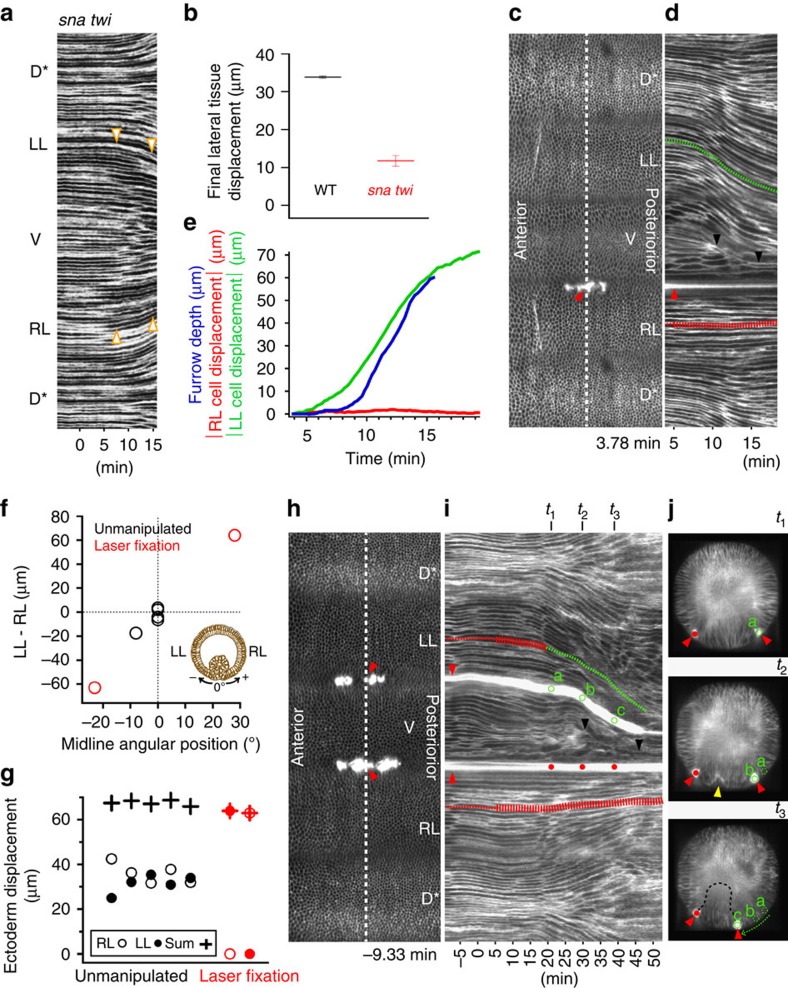
Relationship between ectoderm movement and mesoderm internalization. (**a**) Kymograph of a gastrulating *sna twi* embryo. Arrowheads: positions corresponding to dorsal edges of lateral region in wild-type (WT) embryos. The ‘*' stresses the fact that the same dorsal tissue is represented twice. (**b**) Lateral cell displacement in WT and *sna twi* embryos; >1,200 cells (four embryos) measured per genotype. Bars represent the standard deviation (s.d.). (**c**) Cylindrical projection of embryo with single cauterization (red arrowheads). The ‘*' stresses the fact that the same dorsal tissue is represented twice. (**d**) Kymograph taken along the white dashed line in **c**. Red and green lines mark two individual cell trajectories. Narrow section of the red line: time in which lateral cells in WT embryos are stationary; wide line: the period when WT lateral cells move. Black arrowheads: ventral midline position. (**e**) Furrow depth acell displacement for the embryo in **d**. (**f**) Displacement of lateral cells between 0<*t*<15 min plotted against the final angular position of the midline. (**g**) Final displacements of right (empty circles) and left (filled circles) lateral cell sheets and their sums (crosses). (**h**) Bilateral cauterization (red arrowheads). The ‘*' stresses the fact that the same dorsal tissue is represented twice. (**i**) Kymograph taken along the white dashed line in **h**. Green and red symbols mark the same points and times as in **j**. Dashed lines as in **d** and **j**, Cross-section views of the embryo along the white lines at times *t*_1_, *t*_2_ and *t*_3_ marked in **i**. Red arrowheads as in **h**. Yellow arrowhead: invagination formed at *t*_2_. Black dashed line: outline of the invaginated cell mass. Scale bars, 100 μm.

The displacement of the ectoderm during the internalization of the mesoderm may be entirely caused by the invagination of the mesoderm, or it may be fully or partly independent. To directly assess this, we tested the effects of abolishing furrow formation. We imaged *snail twist* (*sna twi*) double mutants in which the ventral region is transformed from a mesodermal to an ectodermal fate and therefore does not make a furrow (Supplementary Movie 8). Ventral cells do not constrict apically and are not internalized. Nevertheless, from 6 min onwards, the lateral tissue moves ventrally, although the distance it travels is reduced (Fig. 4a,b and Supplementary Fig. 8). These findings rule out the activity of the mesoderm as the sole time trigger and as the exclusive active mechanism for lateral cell behaviour.

Having found that the lateral cells show an activity that is independent of mesoderm internalization, we tested directly their role in mesoderm invagination. We developed a technique to immobilize groups of cells in a defined position by exposing them to an infrared femtosecond laser coupled to MuVi-SPIM for a longer period but with lower power than is used for intracellular laser dissection^17^,^31^ (Supplementary Fig. 9). This treatment cauterizes the tissue and can fix cells to the overlying vitelline membrane (an immobile shell covering the embryo), generating an immobile boundary with a 10–15-μm resolution (Supplementary Movie 9).

Immobilizing tissue along a ventro–lateral line of 80 μm parallel to the AP axis on one side of the embryo impaired the displacement of the lateral cells, causing a displacement of the ventral midline towards the fixed side without stopping the internalization (Fig. 4c–e, Supplementary Fig. 10a–c and Supplementary Movies 10 and 11). Therefore, the central position of the midline depends on both lateral cell sheets moving in synchrony. These findings may help to explain a similar shift occasionally seen in unmanipulated embryos where furrow formation begins at the centre of the ventral side, but the final, invaginated furrow is shifted to one side (Supplementary Movies 12 and 13). The angular position of the midline is affected by the relative speeds of displacement of the two lateral sides (Fig. 4f and Supplementary Movie 11). The left and right displacements can be unequal in some wild-type embryos but the sum of the two displacements remains constant from embryo to embryo. This is true also in embryos in which one side has been fixed (Fig. 4g). This shows that lateral cell sheets are mechanically coupled.

To explore the relationship between the lateral cell populations, we next immobilized both lateral sides simultaneously, with the fixation line positioned between the mesoderm and the ectodermal cell cohort. Furrow formation initiated normally, but phase 2 was disrupted and mesoderm internalization was inhibited (Fig. 4g,h). In the region where the movement of the ectoderm was blocked, the mid-ventral cells remained constricted and the ventro–lateral cells remained stretched, but the furrow did not deepen, whereas in the neighbouring AP regions, furrows appeared (Supplementary Movie 14). This shows that the ability of the ectoderm to move is essential for mesoderm invagination. After 20 min, the fixed tissue on the left detached in this experiment, allowing the movement of the left lateral cells and a delayed invagination (Fig. 4i,j and Supplementary Movie 14).

**Figure 5:**
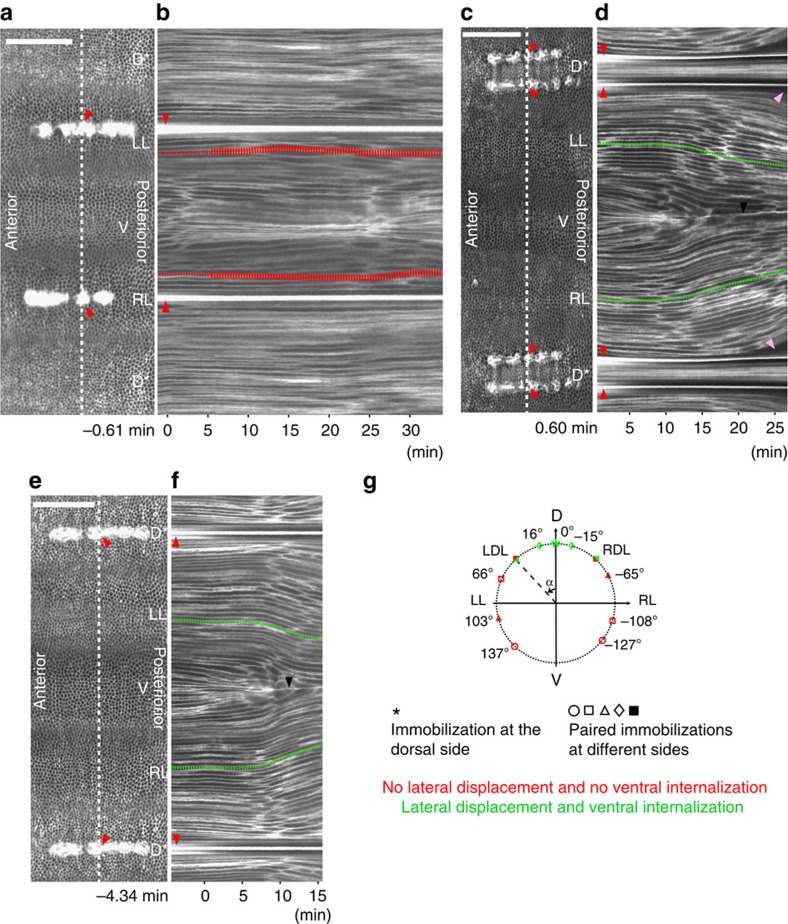
Lateral and dorsal cauterizations. (**a**) Cylindrical projection of an embryo with bilateral cauterizations (red arrowheads). The ‘*' stresses the fact that the same dorsal tissue is represented twice. (**b**) Kymograph taken along the white dashed line in **a**. The red dashed line follows a lateral cell trajectory. (**c**) Cauterizations on the dorsal side of the embryo (embryo **b** in Supplementary Fig. 11). The ‘*' stresses the fact that the same dorsal tissue is represented twice. (**d**) Kymograph taken along the white dashed line in **c**. The green dashed lines follow two lateral cell trajectories. Black arrowhead: ventral midline. Pink arrowheads: detachment of the epithelium from the site of fixation. (**e**) A single fixation at the dorsal midline (embryo a in Supplementary Fig. 11). The ‘*' stresses the fact that the same dorsal tissue is represented twice. (**f**) Kymograph taken along the white dashed line in **e**. Black arrowhead: ventral midline. (**g**) Diagram summarizing the immobilization experiments. Green and red symbols indicate fixations that either allowed or impaired lateral cell displacement and mesoderm invagination. Further cases are summarized in Supplementary Fig. 11. Scale bars, 100 μm.

To test to what extent lateral cell sheet participation is required for invagination, we fixed each ectodermal cell sheet approximately in the middle of the lateral cohort (Fig. 5a). In this condition, phase 1 again proceeded normally, but phase 2 was still disrupted (Fig. 5b and Supplementary Movie 15), showing that when the lateral cell sheet cannot be displaced as a whole unit, none of its cells can move to permit the furrow to internalize. Only when the two immobilizing lines were placed even more dorsally were the lateral cell sheets able to move ventrally and furrow internalization was restored, although with a delay (Fig. 5c,d, Supplementary Fig. 10d and Supplementary Movie 16). In this situation, the dorsal cells were isolated from the lateral sheets. In some cases, the ectodermal cell sheet was eventually torn off the fixed tissue, revealing a ventrally directed pulling force. Finally, when the fixation line was moved to the dorsal midline, gastrulation movements were restored (Fig. 5e–g, Supplementary Fig. 10d and Supplementary Movie 17). To determine the position of the transition between permissive and non-permissive fixations, we conducted a further set of fixations in the dorso–lateral region (note that it is impossible for us to determine *a priori* the precise angular position at which we set the cauterization, since the manipulation is done at a time point before we can see with absolute certainty where the ventral or dorsal midline of the embryo will be). These showed a narrow region at ≈45° from the dorsal midline in which fixations were either permissive or non-permissive, indicating some variation from embryo to embryo (Supplementary Fig. 11). In spite of this apparently sharp border, we do not find a sharp transition between stretched (dorsal) and isotropic (lateral) cell shapes after (Supplementary Fig. 12), but rather a change over a region of 4–5 cells. Plotting aspect ratio and cell orientation against position along the circumference reveals a continuum of cell shapes that can be divided into areas of stretched, oriented cells in the most dorsal region, unstretched but circumferentially oriented cells in the neighbouring region, and unstretched and randomly oriented cells closest to the invaginated furrow. This graded shape change reflects the graded gene expression patterns in this region.

### Mechanical properties of cell populations

**Figure 6:**
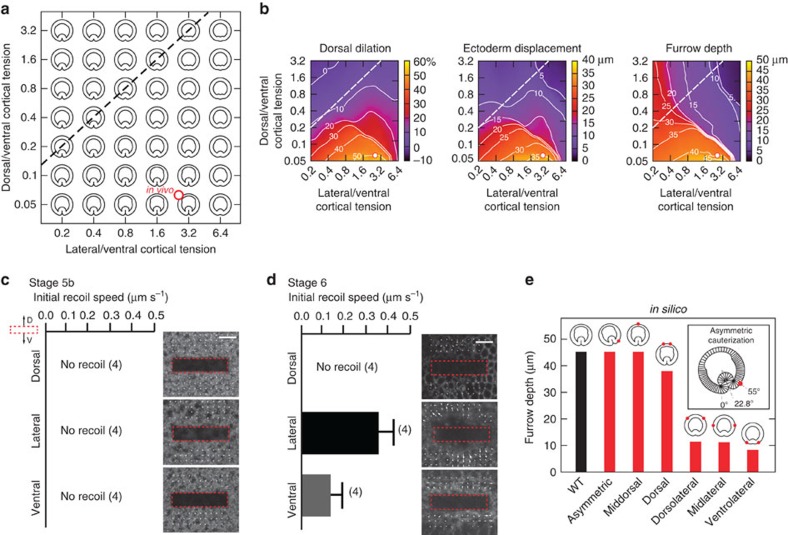
Computational model. (**a**) Phase diagram showing shapes of embryo cross-sections generated by the model with a fixed value of cortical tensions in ventral cells and varying cortical tensions in dorsal and lateral cells. Dashed line connects states with identical cortical tension in lateral and in dorsal cells, and the red circle marks the point where the relative values of lateral and dorsal tension correspond to those measured *in vivo* (**c**). We draw attention to the fact that contrary to the situation in real embryos, the dorsal cells in the model shorten along their apical–basal axis. As the model requires conservation of volume of the cells, and it does not take the third dimension of the cell into account, this is a necessary consequence of cell widening. As we have shown in this work, the cells in fact compensate for widening by shortening in this third, AP dimension. In our model, we accept in this 2D representation the loss of height as a proxy for reduction of the AP length. (**b**) Isolines for values for dorsal dilation, ectoderm displacement and furrow depth achieved with the various combinations of dorsal and lateral tensions shown in **a**. (**c**,**d**) Experimental measurements of the initial speed of recoil of the actin meshwork in ventral, lateral and dorsal regions after laser dissection at a stage before furrow formation (**c**) and at the onset of furrow formation (**d**). (**e**) Shapes and furrow depth generated by the model under different cauterization conditions. Inset shows an enlarged view of the asymmetric case with a displaced furrow.

The above results show that the different cell populations along the DV axis have different properties, and they suggest that these properties determine how the cells behave during early gastrulation. These findings can be used to elaborate the theoretical understanding of furrow formation and invagination. We used as starting point our previously developed computational framework in which cell behaviour is determined exclusively by cortical tension in an epithelium of identical cells^32^, and generalize this model by defining three subpopulations (ventral, lateral and dorsal) with different mechanical properties (Supplementary Fig. 13). The cross-sectional area of all cells is set as constant. The onset of furrow formation is triggered by an increase of apical tension in ventral cells, which leads to their apical constriction. The deformation response of the rest of the blastoderm then depends on the cortical tension of the lateral and the dorsal tissue measured relative to that in ventral cells. This effect is best represented by the phase diagram of the minimum-energy final embryo shapes (Fig. 6a,b). The deformation of each cell is inversely proportional to its effective Young modulus, which scales with its cortical tension (see equation (2) in Methods section). The simulations show that a differential modulus of the lateral and dorsal tissue is crucial to reproduce embryos with the correct dorsal widening, lateral cell displacement and furrow depth (Fig. 6a,b). States with identical moduli of lateral and dorsal tissue result in too small an average ectoderm displacement and dorsal dilation. More specifically, our model predicts that the cortical tension in lateral cells should be ≈3 times the cortical tension in ventral cells, and that the cortical tension in dorsal cells is at least two orders of magnitude smaller than that in ventral cells.

To test the model's quantitative predictions of tension *in vivo*, we performed infrared laser dissection of the apical actomyosin meshwork where initial recoil speed of the meshwork can be used to infer the cortical tension^33^,^34^. We compared tensions in three regions of the embryo before and after the beginning of gastrulation. Before gastrulation (stage 5b), the actin meshwork does not recoil after laser dissection in any of the locations tested (Fig. 6c). At the onset of gastrulation (stage 6), the actomyosin meshwork shows different behaviours in the three regions. We observe no recoil in dorsal cells, fast recoil in lateral cells and slow recoil in ventral cells (Fig. 6d). The measured relative cortical tensions fall in the region of the phase diagram where our model reproduces the overall deformation behaviour in wild type (Fig. 6a,b). In addition, the recoil of ventral tissue lasted for longer (4.0±0.8 s) than that of the lateral tissue (<1 s) (Supplementary Movies 18 and 19, and Supplementary Fig. 14), showing that the latter is stiffer than the former.

To further test the model, we compared the theoretical deformation behaviour in manipulated embryos with *in vivo* observations. With the above relative cortical tensions, the model correctly reproduces all results of bilateral cauterization experiments and the displacement of the ventral midline after unilateral cauterization (Fig. 6e), but if the mechanical differences are either disregarded or inverted it fails (Supplementary Fig. 13g). This further confirms our quantitative prediction of relative cortical tensions, and, surprisingly, suggests that (i) the complex behaviour of both wild-type and manipulated gastrulating embryos can be described by a small number of parameters, and that (ii) the differential mechanical properties and tissue deformation play a key role in defining how the embryo behaves during the first phase of gastrulation.

## Discussion

Genetic and molecular evidence indicates that the forces that create the ventral furrow and lead to the invagination of the mesoderm are generated within the mesoderm. Our results show that other parts of the embryonic body contribute to the dynamics and timing of the process. This had not been possible to deduce from studies using fixed materials or more localized live observations.

We demonstrate interdependencies of epithelial movements during the early stages of gastrulation. Mesoderm internalization is always temporally correlated with lateral cell displacement. The timing of the two processes is not strictly linked to the timing of ventral apical constriction: apical constriction is neither sufficient nor necessary to set the timing. Apical constriction alone also does not define the position of the ventral midline at which the two ectodermal cell sheets meet, since the relative movements of the lateral cell sheets play a role in its central positioning. Finally, lateral displacement is not exclusively dependent on mesoderm internalization, but is necessary for it.

Dorsal cell widening appears to be largely passive and the consequence of an active ventrally directed force transmitted through the neighbouring tissue. We conclude this from the finding that after cauterizations near the dorsal edge of the lateral ectoderm, the tissue ventral to the immobilization site can tear itself off the wound, indicating a ventral pulling force. In addition, if an active dorsal process was at work we would expect to see dorsal buckling along the DV axis after lateral cauterization.

The cells along the DV axis do not behave in an identical manner, and in particular they have distinct mechanical properties. In the mesoderm, the central cells constrict and the peripheral cells stretch. In the ectoderm, the lateral cells behave as a stiff, non-deformable unit, whereas the dorsal cells act as a softer and stretchable unit. Myo-II can regulate the stiffness and contractility of F-actin meshworks^35^,^36^. The differences in the mechanical properties of the cell populations in the *Drosophila* embryo are reflected in differences in their cytoskeletal scaffolds, which suggest that the cytoskeleton has a role in mediating the observed differences in behaviour. This is true especially for the medial–apical actomyosin meshwork, which has been shown to be linked across cell boundaries and to control cell shape^2^,^4^,^9^,^16^.

The model we present was developed to make predictions about the surface tensions of cells that are necessary to generate the tissue movements we observed *in vivo*. It made the surprising prediction that to generate the tissue movements we observe *in vivo*, the highest tensions were needed in the lateral tissue and lowest in the dorsal cells, properties that were then experimentally shown to pertain *in vivo*.

The model does not recapitulate perfectly all cell shape changes seen in cross-sections of real embryos. This has already been addressed, and achieved more accurately, by other models^25^,^37^. We therefore accept the two-dimensional (2D) representation of volume conservation as a proxy for 3D volume conservation, even though it leads to representations that do not correspond to the shapes of cells in the real embryo, as is the case for the dorsal cells in our model. Specifically, when dorsal cells in the model widen, they reduce their apical–basal length at the same time. This does not reflect the situation *in vivo*. It is a consequence of the model representing cells in only two dimensions and the cross-sectional surface of cells being set as constant. As we have shown above, dorsal cells in real embryos compensate the widening in the circumferential direction by shortening in the AP direction, a change that the model cannot recapitulate. Future technical developments should allow us to analyse single-cell properties in 3D at greater resolution and depth to feed computational models that better represent real cell shapes and test more sophisticated predictions.

While we do not yet know the underlying mechanisms for the differential mechanical properties, the differences in the subcellular architecture of cells along the DV axis must be connected to the cells' fates. For example, the contractile actomyosin meshwork that accumulates apically in the central region of the mesoderm depends on the Twist target genes Fog and T48 (refs 5, 6, 27, 38), which are expressed just prior the time and in the region, and in which the apical constrictions take place (Supplementary Fig. 5a). At the same time, all mesodermal cells disassemble their adherens junction under control of the transcription factor Snail^6^ (and they reassemble them apically), which distinguishes them from the neighbouring ectodermal cells. The later apical relocation of junctions and actomyosin assembly that occurs in the ectodermal cells as they displace ventrally is controlled by the JAK/Stat pathway^39^ and an ectoderm-specific inhibition of the ubiquitin-ligase neuralized^40^. In future, this knowledge of the control of cell biology can be used to directly manipulate actomyosin networks to test how they are integrated at the embryo scale to enable gastrulation.

## Methods

### Fly strains and crosses

The following fly stocks were used in this study: *sqh-Gap43::mCherry*/CyO was used to label membranes (kindly provided by Stefano De Renzis), *w;;sqh-MoeABD::*eGFP (Dan Kiehart, Duke University, Durham, NC) was used to image F-actin and *sqh*^*AX3*^*; sqh-MRLC::GFP* (II) to visualize Myo-II. To image *sna twi* mutant embryos, the amorphic alleles Df(2L)TE116GW11 (Df(*sna*)) (ref. 41) and Df(2R)*twi*^*S60*^ (ref. 41) were recombined to *Δhalo*^*AJ*^ (ref. 41), a recessive mutation in the *halo* gene, which produces a visible phenotype in yolk clearing (41). Homozygous mutant embryos were selected from eggs laid by *Δhalo*^*AJ*^ Df(2L)TE116GW11 Df(2R)*twi*^*S60*^/CyO; *UASp-Gap43::mCherry*/MKRS flies (gift from Martina Rembold and Thomas Lecuit). Ventralized embryos expressing markers for membranes and Myo-II were obtained by first mating *Tl*^*10B*^/TM3 male flies to *sqh*-*MRLC*::eGFP/Cyo;*UASp-Gap43::mCherry/MKRS* female flies. This cross produces female flies that are heterozygous for the dominant *Tl*^*10B*^ allele (*sqh*-*MRLC*::eGFP/+; *Tl*^*10B*^/*UASp-Gap43::mCherry*), which were crossed to *sqh*-*MRLC*::eGFP/Cyo;*UASp-Gap43::mCherry/MKRS* males to collect ventralized embryos. The stock *sqh-Resille::mCherry; bnt/TM3* was a gift from Eric Wieschaus.

### MuVi-SPIM imaging and data processing

Imaging was performed using a MuVi-SPIM^23^. Illumination was done using Nikon × 10/0.3w objective lenses and detection with Nikon × 25/1.1w objective lenses, resulting in an effective image pixel size of 0.26 μm × 0.26 μm. Optical sections of an embryo were recorded with a typical spacing of 1 μm. Therefore, the effective volume of every voxel was ∼0.07 μm^3^. Embryos were imaged from two opposing directions simultaneously and successively from two directions normal to the first two (0°=dorsal and ventral view; 90°=lateral views), with time resolution ∼15–20 s per complete data set. Therefore, for every time point, we collected four 3D images of the embryo.

The four image stacks of every time point were combined into a single isotropic image (identical sampling along all three axes). An outline of the basic protocol is described in Supplementary Fig. 1 with details provided below. First, the two views for each rotation were fused into a single image using an affine transformation derived from beads taken after every imaging session^23^. Note that these intermediate images were not isotropic (typical voxel size was 0.26 μm × 0.26 μm × 1 μm). Second, the intermediate 90° image was transformed into the same coordinate system as the intermediate 0° image. This process involved image-based alignment and consisted of the following steps:

- The two intermediate images for the first time point were downscaled by a factor of four in *x–y* plane (so typical voxel size was 1.04 μm × 1.04 μm × 1 μm).
- Beads were used to estimate the initial affine transformation.
- Image-based alignment^42^ was used to optimize the image alignment of the two downscaled images (note that the code for performing this operation was significantly sped up from that published previously^23^ through enabling non-isotropic images to be handled).
- The optimized affine transformation parameters were then used after appropriate rescaling of the translations as an initial guess for aligning the full-size intermediate images using image-based alignment.
- Owing to mechanical stability of MuVi-SPIM, full affine parameters only had to be determined for the first time point and could then be reused for the subsequent time points, optimizing only the translation part of the transformation. By restricting the free parameters to only linear translations (that is, just three degrees of freedom) the image-based alignment was fast (few minutes per time point on a computer with 3.33 GHz clock, 12 cores and 192 GB SDRAM) even for full-size images, which were typically each ∼750 MB.
- After transforming, the intermediate 90° view, the images were made isotropic by scaling the *z*-direction to 0.26 μm pixel size using linear interpolation and then fused into a single image^23^. The final fused image for each time point had an isotropic resolution of 0.26 μm in all directions (isotropic image ∼3 GB in size). To fully process a movie of 150 time points at full resolution typically took ∼1 day of computation on the workstation described above. This process was robust, with sub-micrometre level of precision in embryo alignment around the embryo centre, as necessary for analysis of membrane-localized signals in the region of interest considered in the paper. For processing data from the laser immobilization experiments, an additional complexity arose from the high fluorescence present at the exposed region (typically 5–20 times brighter than the Gap43::mCherry membrane signal). For these embryos, the alignment worked well after using an upper intensity threshold (typically set to ∼50% higher than the mean membrane-localized mCherry signal) to remove these very bright regions during image-based alignment (otherwise the regions exposed to infrared laser heavily biased the image-based alignment algorithm).

Once the data were aligned and fused, we performed unrolling to extract the apical surface of the embryo. The process of unrolling involved two main steps. First, the embryo surface was segmented in three dimensions to identify the embryo outline. This had to be done with high precision as small errors were detrimental to the subsequent unrolling. The segmentation processing scheme is outlined in Supplementary Fig. 2a. Note that for embryos exposed to infrared laser, manual correction of the final mask was required to correct for distortions caused by the very high intensity in the exposed regions. Second, the segmented image was projected into two dimensions^23^,^43^. Here we discuss the particular methodology used to represent our data. For each point ([*x*,*y*,*z*]) on the surface of the embryo segmentation, the appropriate spherical coordinates ([*r*,*θ*,*ϕ*], where *z*=*r* cos*θ, x*=*r* cos*ϕ* sin*θ* and *y*=*r* sin*ϕ* sin*θ*) were found (Supplementary Fig. 2b). We then found a representation of the embryo surface using spherical harmonics^44^,^45^. Briefly, the use of spherical harmonics allows the representation of the embryo surface to be completely parameterized by two angles *θ* and *ϕ* (Supplementary Fig. 2b), where the radius from the embryo centre to a particular point **r**=**r**(*θ*,*ϕ*). Typically, 20 degrees of spherical harmonics were sufficient to smoothly represent the embryo surface at all positions. The embryo surface represented by spherical harmonics is demonstrated in Supplementary Fig. 2c. A major advantage of using the spherical harmonic representation is that finding the direction normal to the surface at all positions is quick and accurate, as we have an analytical expression for the normal Supplementary Fig. 2d. Numerically calculating the normal direction from the surface using the segmented image has relatively large local errors due to fluctuations that are smoothed out by the spherical harmonic representation. Using these normal vectors, we can shrink the surface to different depths from the surface of the embryo (for example, we can find an inner surface for which all points are 20 μm from the apical surface of the embryo; Supplementary Fig. 2e. We typically found inner layers up to a depth of 20 μm from the apical surface using 1 μm steps along the direction of the normal vectors. For each layer, we performed a cylindrical projection onto a 2D sheet (Supplementary Fig. 2f)^23^. Our primary focus for this work was the 180-μm-wide region along the AP axis at the centre of the embryo. This region was only minimally distorted by the cylindrical projection, allowing straightforward calculation of cell properties such as surface area^23^.

### Optical sections of the embryo

Cross, sagittal and coronal sections were obtained using Fiji software, and selecting one plane out of a 3D-fused stack. All cross-sections are taken at half embryo length.

### Cell segmentation tracking and analysis

Projected surface views of cells were segmented and tracked with the freeware software Ilastik^46^ and Cell-Profiler^47^ respectively. Cell shape analysis and cell kinematics were measured using CellProfiler and Matlab. Track rendering was obtained with Imaris (Bitplane) software. The internalization of the ventral furrow was tracked by using the ‘Manual Tracking' Fiji plugin that also provided measures of the furrow depth. Imaris (Bitplane) software was used for rendering. Cell orientation (for example, Fig. 3c,d) is defined by fitting an ellipse to the cell and measuring the angle of the major axis. The axis parallel to the AP axis of the embryo is defined as 90°, the axis parallel to the DV axis as 0°, as illustrated in the schematics (Fig. 3d, bottom, and Supplementary Movie 12). The *y* axis in the plots is thus cell orientation (average and s.d.), whereas the colour shows the aspect ratio (ratio between major and minor axis of the fitted ellipse to cells).

### Embryo time alignment

We considered as time ‘0' the point at which the ventral cells had reduced their apical cells surfaces by an average of 20%. To align wild-type and mutant embryos, we used the following three other developmental processes as temporal markers: (i) the depth of membrane invagination during cellularization in dorsal cells reaching 30 μm; (ii) the initiation of cephalic furrow formation; and (iii) the edge of the PMG having been displaced on the dorsal side by a distance equal to ¼ of the embryo length towards the anterior. For *sna twi* embryos, that lack apical constriction, we measured only three parameters and minimized the time distance of these three time markers compared with wild type. In *Toll*^*10*^ mutants, only cell lengthening during the fast phase of cellularization can be used, and this measure was aligned with the corresponding measure in wild-type embryos. In *bnt* mutants, only 20% of ventral cell surface reduction and cell lengthening during cellularization were used (Supplementary Fig. 3).

### Local tissue immobilization

Local tissue immobilization can be performed by exposing the tissue to an infrared femtosecond laser (Mikan, Amplitudes Systèmes) at an average power below and time exposure above what is used to perform plasma-induced ablation^48^. Local laser exposure of the *Drosophila* blastoderm epithelium to such irradiation presumably results in the fusion of the tissue with the overlying vitelline membrane. Since the vitelline membrane is immobile, the fusion results in the creation of a fixed obstacle in the tissue. This obstacle can be more or less tightly fixed depending on the *z*-focus position of the objective. By focusing the laser at the interface between epithelium and vitelline membrane, it is possible to generate a fixed obstacle that will not move throughout the process of mesoderm invagination. Focusing the laser 2–5 μm below the surface of the tissue generates obstacles that will resist to a certain extent the tissue movement but will then detach, allowing the tissue to resume more typical wild-type behaviour.

We performed local tissue immobilization experiments with a custom-built system. A near-infrared (NIR, 1,030 nm) femtosecond laser at 50 MHz repetition rate (Mikan, Amplitude Systèmes) was coupled to the MuVi-SPIM. Tissue immobilization and MuVi-SPIM 3D time imaging were performed in series. The NIR-femtosecond laser beam is expanded through a × 15 telescope and is aligned with the microscope optical path with a dichroic mirror immediately before one of the two detection MuVi-SPIM objectives lenses ( × 25/1.1 numerical aperture (NA), water immersion, infrared corrected, CFI Apo LWD, Nikon). The NIT-femtosecond beam is expanded in a way that it fills the back aperture of the objective lens that transmits 63% of the incoming NIR light. Local tissue immobilization is achieved by exposing the apical side of the epithelium, as close to the vitelline membrane as possible, to 200 mW average laser power for 40 ms at the back aperture of the objective. Performing a local tissue immobilization along a line of 150 μm took 12–20 s, depending on the number of manual refocusing procedures that were required because of the slight curvature of the embryo.

### F-actin and Myo-II fluorescence imaging

We imaged *MoeABD::GFP* (see fly stocks) as a proxy for F-actin and MRLC (myosin relative light chain) GFP using a spinning disc confocal system (PerkinElmer) with a × 63/1.2 NA water immersion Zeiss objective.

### Actomyosin meshwork ablation

We performed laser-based actomyosin meshwork ablation by using a femtosecond-pulsed infrared laser (Chameleon Compact OPO Family, Coherent) tuned at 950 nm and coupled into a LSM Zeiss 780 confocal microscope. We used the Zen Bleaching interface to create the region of interest and set the laser power to 65% with one single cycle of exposure. For this experiment, we used the C-Apochromat × 63 water immersion Zeiss Objective with 1.2 NA (infrared corrected).

### *In silico* modelling

In the 2D implementation of our mechanical model, we consider the cross-section of the developing embryo consisting of a ring of *N*=80 cells, where each cell is represented by a quadrilateral with straight edges. Both cell interior and yolk are approximated by incompressible fluids so that the volume of each cell and the yolk volume are fixed. These constraints imply that the cross-section area of each cell *A*_c_ and of yolk *A*_y_ are fixed too; based on experiments we choose *A*_c*/*_*A*_y_=11/600=0.0183 (ref. 32). Apart from being subject to these constraints, cell shape is governed by intracellular forces due to the contractility of actomyosin cortical network and cell–cell adhesion mediated by cadherins. We introduce effective surface tensions of the apical, basal and lateral cell sides Γ_a_, Γ_b_ and Γ_l_, respectively, which are all different from each other. The total energy reads:


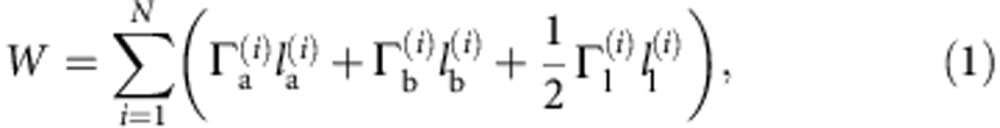


and the minimal-energy shape of the embryo is computed using Surface Evolver package^49^.

Apart from the intrinsic preferred cell shape, which is given by the model parameters as studied in ref. 50, and the fixed-yolk-area and fixed-cell-area constraints, the overall shape of the epithelium is also determined by the vitelline membrane. We model it as a circular cavity with the resting radius 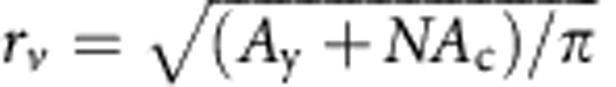 (ref. 32).

The shape of an epithelium containing more than a single type of cell depends on the cells' differential elastic moduli. In particular, the Young modulus of a cell is controlled by the relative tensions *α*=Γ_a_/Γ_l_ and *β*=Γ_b_/Γ_l_, as well as by cortical tension *σ*=(Γ_a_+Γ_b_)/2 (ref. 50) and reads:


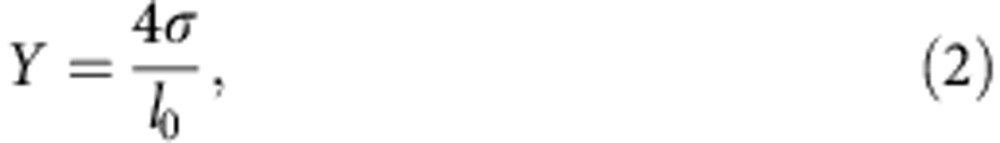


where 
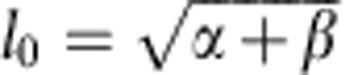
 is the preferred cell height^50^. Cells of identical shape have the same relative apical and basal tensions *α* and *β*, but their cortical tensions *σ* and thus their Young moduli *Y* may differ. In stiff cells, the magnitudes of the three tensions Γ_a_, Γ_b_, Γ_l_ and the cortical tension are larger than in soft cells. We choose the cortical tension in ventral cells as a unit tension and measure all other tensions relative to it (Fig. 6a,b).

The reduced-dimensionality implementation of the model has several advantages and is a common practice in studying problems of cylindrical symmetry like early-stage *Drosophila* embryo. This approach relies on fewer parameters and does not involve the topological structure of the tissue, which is subdominant in epithelial folding. As a result, it allows us to expose as clearly as possible the main novel aspect of the mechanics at work. The main simplification made is of geometrical nature: In the 2D implementation, an increase or decrease of cell cross-section width necessarily leads to a change of cell height, whereas in the true 3D model a part of this deformation would be transferred to a change of the lengthwise dimension of the cell. In addition, the relative magnitude of the lateral surface energy compared with the apical and basal surface energies is smaller because of geometrical reasons; this effect may be compensated by rescaling the tensions. Finally, we stress that the main driving force for ventral furrow formation is apical constriction in ventral cells due to differential apico–basal tension, which is present in both 3D and 2D implementation, rendering our model suitable for the problem at hand.

*Building a model wild-type embryo*. In zero-order approximation, the embryo consists of *N*=80 identical cells arranged on a ring. The model represents the process of ventral furrow formation as a trajectory in phase space driven by a gradual temporal variation of apical tension in 10 ventral cells. Specifically, ventral cells constrict apically due to an increase of their apical tension (Supplementary Fig. 13a,c). The final differential apico–basal tension Γ_a_–Γ_b_=3Γ_l_ in ventral cells is large enough so that ventral furrow formation is completed for the appropriate values of cortical tension in lateral and dorsal cells (Fig. 6a).

To study the differential mechanics of the three cell populations, we explore the dependence of the embryo cross-section on cortical tensions in lateral tissue and dorsal tissue (Fig. 6a), focusing on the following three key observables: (i) dilation of the dorsal tissue, (ii) average displacement of lateral cells and (iii) furrow depth (Fig. 6b). To further restrict the relevant ranges of cortical tensions, we perform a theoretical version of the cauterization experiment isolating dorsal tissue and part of the lateral tissue from constricting ventral cells (Supplementary Fig. 13b). This study shows that if the cortical tension in the lateral cells is large enough, the lateral cells in the vicinity of constricting ventral cells are unable to deform, and therefore furrow cannot fully internalize.

*Detailed spatial profile of apical tension*. In the above discussion, it is assumed that the apical tension profile is a simple step function (Supplementary Fig. 13a). It is however not clear how the details of the profile affect the final shape of the embryo. Here we show that the formation of ventral indentation due to simultaneous apical constriction of all 10 constricting cells can be achieved by a tension profile where the apical tension in the centre of the ventral segment increases faster than in the cells contiguous with the lateral cells (Supplementary Fig. 13c,d). Such a profile is consistent with the known graded distribution of the inducers of furrow formation (Supplementary Fig. 5b).

*Surface tensions variation mimics furrow formation dynamics*. The trajectory from the initial state to the final state is nicely illustrated by a simulated best-fit theoretical kymograph shown in Supplementary Fig. 13e, which reproduces most features of the experimental kymograph (Fig. 3a); here the time dependence of the increase of apical tension in ventral cells was given by a power law (specifically, *t*^4^). Coloured arrows indicate the distance between two chosen cells from the same population and reveal the different deformation modes characteristic of the three cell populations: apical constriction of ventral cells, dilation of dorsal cells and displacement of lateral cells.

*Vitelline membrane*. The pressure exerted by the vitelline membrane is given by *p*(*r*)=*p*_0_{exp[(*r*−*r*_*v*_)/*r*_*v*_]−1}, where *r*_*v*_ is the resting radius of the vitelline membrane and *r* is the radial distance from the centre of the embryo. Pressure here is measured in the units of 
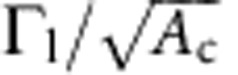
. We can treat the vitelline membrane as very stiff by choosing an arbitrary, high but still finite value of *p*_0_. We choose *p*_0_=1,000; qualitatively, the result for a semi-hard membrane (*p*_0_=10) is not different (Supplementary Fig. 13f).

*Modelling cauterization experiments*. To further examine the differential behaviour of the three cell populations, we model all fixation experiments with two additional sets of parameters clearly distinct from our best-fit ratio of cortical tensions of 1:2.7:0.075 (Fig. 6e and set I in Supplementary Fig. 13g). In the first modified set, we swapped the cortical tensions of the dorsal and the lateral cells, making the former very rigid and the latter very soft; the ratio is thus 1:0.075:2.7 (set II in Supplementary Fig. 13g). In the second modified set, the cortical tension of all three cell populations is the same, the only difference between them being that the ventral cells still undergo apical constriction (set III in Supplementary Fig. 13g). Supplementary Figure 13g shows the furrow depth for all three parameter sets in wild-type embryo and for the six different cauterization experiments, and it is clear that both modified parameter sets II and III predict a wrong deformation behaviour of the cauterized embryos. This further confirms our prediction of the ratio of cortical tensions and the corresponding elastic properties of the three cell populations.

## Additional information

**How to cite this article:** Rauzi, M. *et al.* Embryo-scale tissue mechanics during *Drosophila* gastrulation movements. *Nat. Commun.* 6:8677 doi: 10.1038/ncomms9677 (2015).

## Supplementary Material

Supplementary Information Supplementary Figures 1-14 and Supplementary Reference

Supplementary Movie 1 Time-lapse of a cylindrical projection and cross-section view of a Drosophila embryo during early gastrulation extracted from 3D data sets at each time point.

Supplementary Movie 2 Time-lapse of a cylindrical projection in which cells of the entire embryo have been tracked.

Supplementary Movie 3 Segmentation and tracking of the ventral constricting cells.

Supplementary Movie 4 Manual tracking of the apex of the internalizing furrow.

Supplementary Movie 5 Time-lapse illustrating the accumulation of Myo-II clusters in the lateral cell population before and during its displacement towards the ventral side of the embryo. Red: E-cad; green: Myo-II.

Supplementary Movie 6 Time lapse showing displacement behaviour of lateral cells.

Supplementary Movie 7 Time-lapse showing the central cross-section of a wildtype embryo and of a bnt embryo during mesoderm invagination.

Supplementary Movie 8 Time-lapse showing a cross-section of a sna twi embryo during gastrulation.

Supplementary Movie 9 Detail of a time-lapse cylindrical projection over ~20 min of gastrulation showing a cauterisation in the lateral region of the embryo.

Supplementary Movie 10 Time-lapse showing a cylindrical projection of a Drosophila embryo during gastrulation on which a single cauterisation was performed on the right, ventro-lateral side of the embryo.

Supplementary Movie 11 Time-lapse of the cross-section of an embryo. The black spot corresponds to the region of fixation.

Supplementary Movie 12 Predicted mid-line position during ventral tissue internalization in a wildtype embryo.

Supplementary Movie 13 Predicted mid-line position during ventral tissue internalization in an abnormal wildtype embryo.

Supplementary Movie 14 Time-lapse of a cylindrical projection of an embryo with bilateral cauterisations in ventro-lateral positions.

Supplementary Movie 15 Time-lapse of a cylindrical projection of an embryo with bilateral cauterisations.

Supplementary Movie 16 Time-lapse of a cylindrical projection of an embryo with cauterization on the dorsal side of the embryo.

Supplementary Movie 17 Time-lapse showing a cylindrical projection of an embryo with two dorsal cauterisations.

Supplementary Movie 18 IR laser dissection and recoil of the F-actin meshwork on the lateral side of the embryo.

Supplementary Movie 19 IR laser dissection and recoil of the F-actin meshwork on the ventral side of the embryo.
