# Supplementary material for: Embryo-scale tissue mechanics during Drosophila gastrulation movements
Source: Nat Commun. 2015 Oct 26;6:8677. doi: 10.1038/ncomms9677 (PMC4846315; doi:10.1038/ncomms9677)
Supplement: Supplementary Information — Supplementary Figures 1-14 and Supplementary Reference [file ncomms9677-s1.pdf]

Supplementary Figures

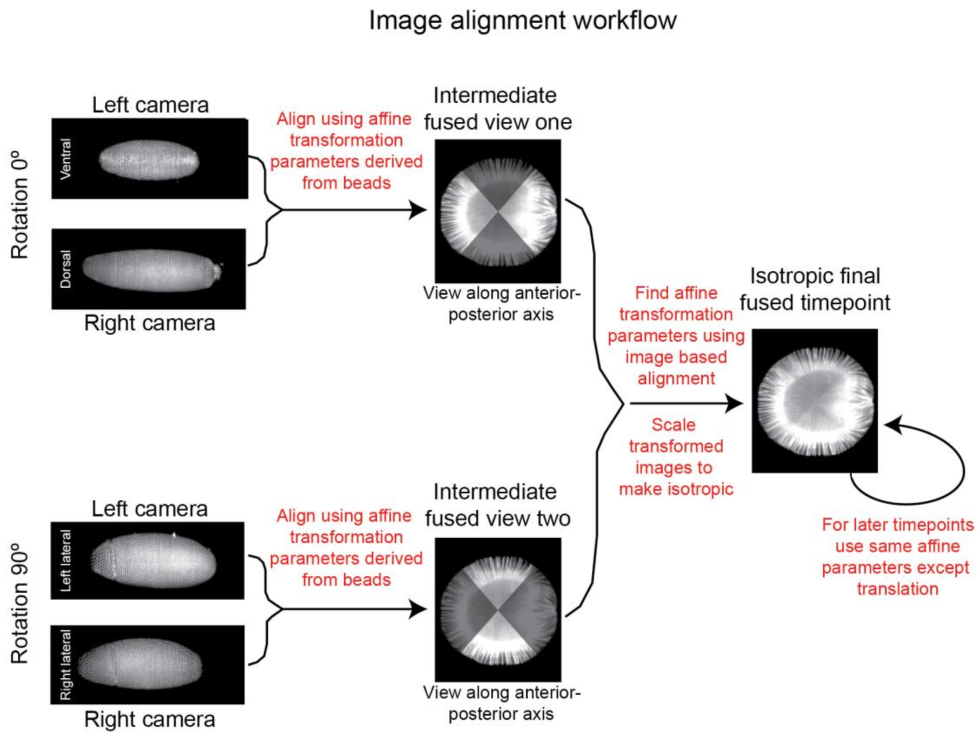

Supplementary Figure 1 │ Image alignment and fusion. Workflow chart for image alignment and fusion for one time point. Details of specific components can be found in the supplementary materials. Images are a visual aid, taken from a fully fused isotropic embryo. For the left column, representative planes are shown of where each view (camera and rotation combination) produced the highest quality data. For the intermediate fused images, the shading is used to qualitatively demonstrate the contributions to the image from different cameras and rotations.

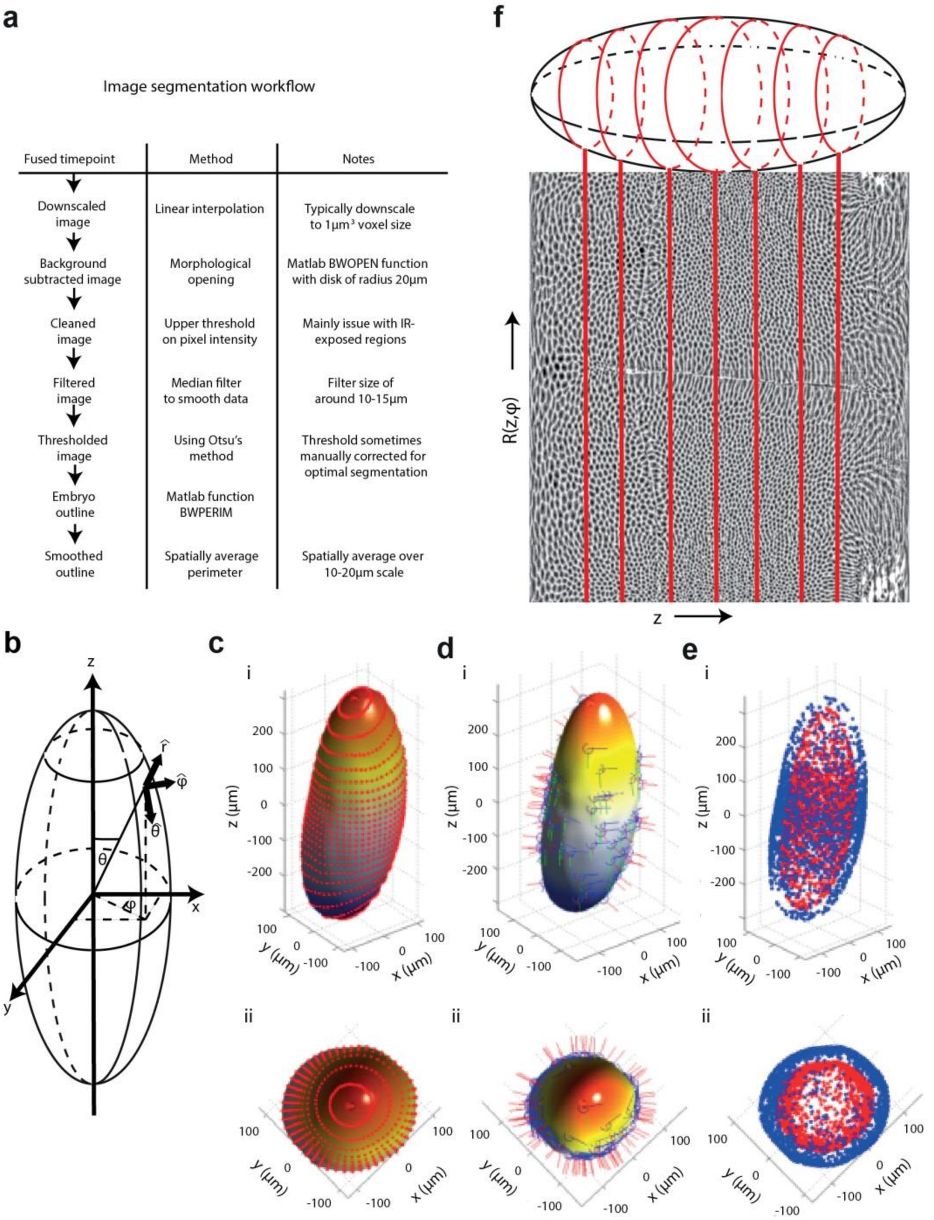

Supplementary Figure 2 │ Image segmentation and 2D data projection. a, Workflow chart for segmentation of the embryo outer surface after image fusion. All processing was performed in Matlab. **b**, Spherical polar coordinate system (r, θ, φ) compared to Cartesian coordinates (x, y, z). θ defines the polar angle and φ the azimuthal angle. **c**, Embryo surface defined by spherical harmonics (colour represents position along antero-posterior (AP) axis: red and blue represent the anterior and posterior poles respectively). Each closed loop formed by the red dots denotes a region of equal θ. **d**, Embryo representation as in panel c. Red arrows denote normal vectors to surface for 100 randomly selected positions on the surface. Green and blue arrows denote orthogonal vectors lying parallel to the embryo

surface. **e**, Blue points represent randomly selected positions on the embryo surface (after smoothing using spherical harmonic representation). Red points represent an inner layer where each position is 20 μm from the surface. In **c-e**, (i) shows a side view of the embryo and (ii) the view along the AP axis seen from the anterior pole of the embryo. **f**, Schematic of the cylindrical unrolling of the surface layer of the embryo onto a two-dimensional sheet. z represents the distance along the AP axis and remains unchanged by the projection. $\mathbf{r}(z, \varphi)$ represents the distance around the embryo for each z position. The cylindrical transformation is chosen such that at the embryo centre the pixel size in the unrolled view is 0.26 μm x 0.26 μm. Toward either pole the effective pixel size decreases in $\mathbf{r}(z, \varphi)$, resulting in stretched cells in the unrolled projection toward both poles.

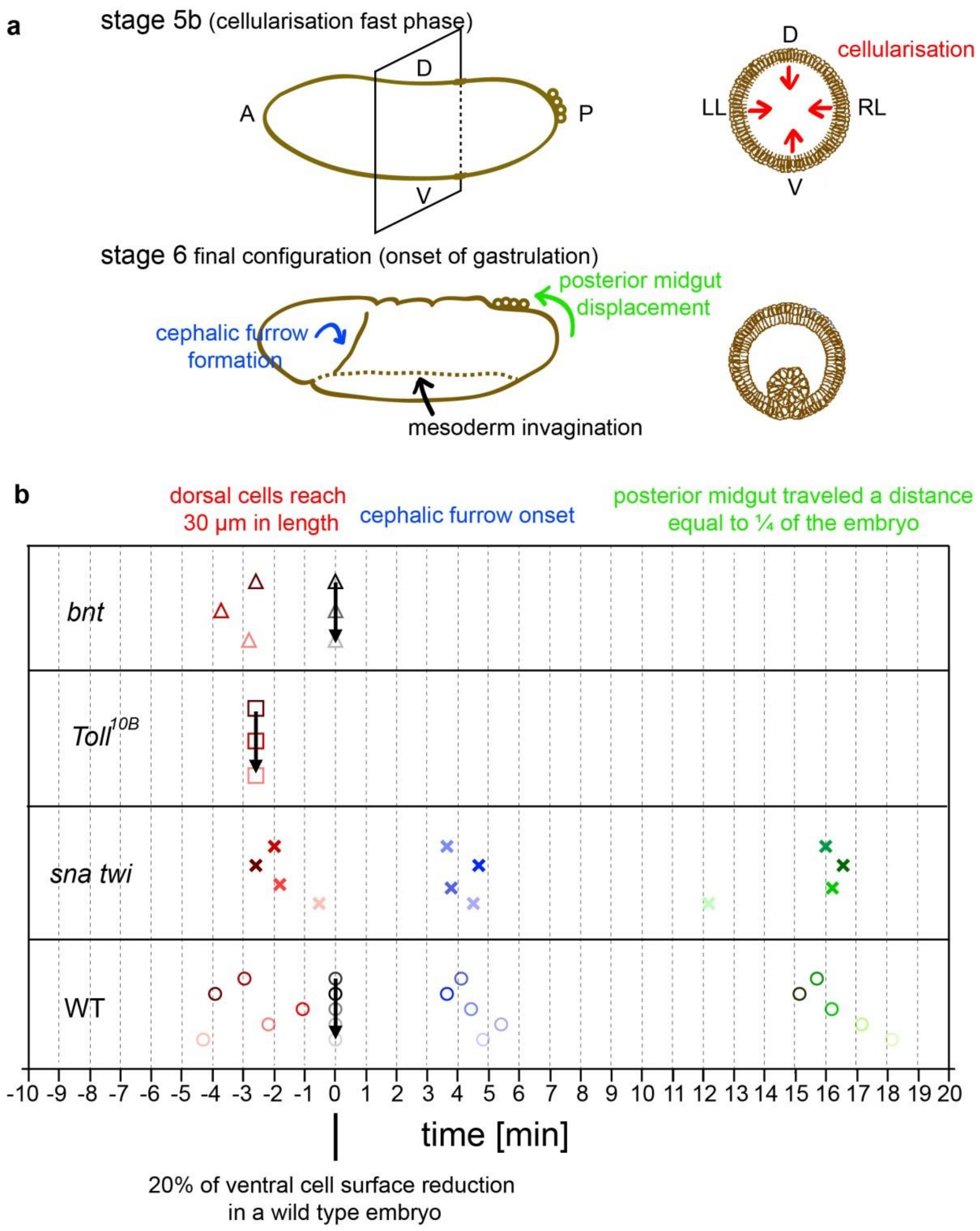

Supplementary Figure 3 │ Temporal alignment of embryos by morphogenetic events. a, Cartoon showing the four major morphogenetic processes taking place just before and during the initial phase of gastrulation. **b**, Diagram showing the time points of morphogenetic events in wildtype and mutant embryos. Each horizontal line of symbols represents one embryo. The time points at which the following events took place were recorded: the cellularisation front in dorsal cells had reached a depth of 30 µm; the first signs of the cephalic furrow were visible; the edge of the posterior midgut invagination had moved up to 1⁄4 of the embryo length on the dorsal side; ventral cells had reduced their surface by 20% on average. The time for the latter

event was then set as t = 0. Not all of these events occur in all mutants. The mutant timing was therefore aligned to the wildtype timing by minimizing the differences (*sna twi*) or using the time points of 20% ventral cell surface reduction (*bnt*) or cellularisation (*Toll*^{10B}).

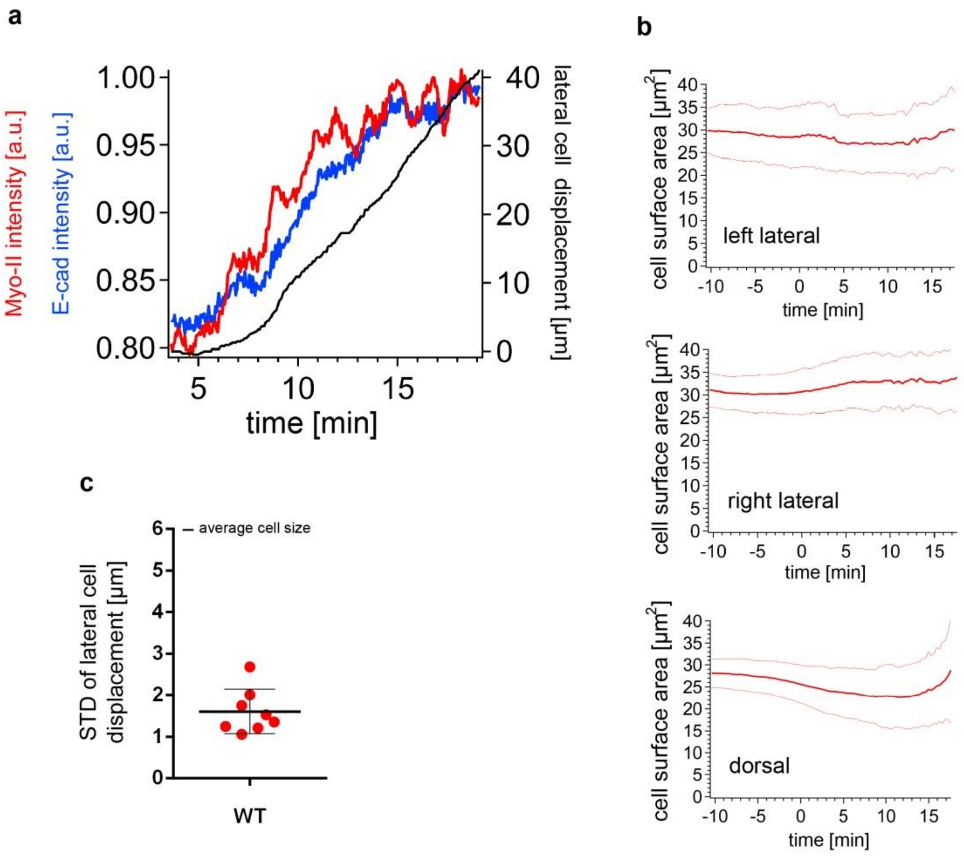

Supplementary Figure 4 │ Lateral tissue properties. a, Graph showing extent of lateral tissue displacement and normalized Myo-II and E-cad average intensities on the apical side of lateral cells as a function of time (refer to Supplementary Movie 15). The level of activated, apically located Myo-II in the ectoderm appears to increase in a stepwise manner. **b**, Average and standard deviation of surface area of cells in the left lateral, right lateral and dorsal regions as a function of time. **c**, Standard deviation of cell displacement along the DV axis for eight groups of lateral cells in four embryos. Cell displacement was measured for ≈ 150 lateral cells on each side of four embryos, and the standard deviation calculated for each group. The average of the standard deviation for the eight groups is 1.6 µm, approximately a quarter of a cell diameter (≈ 6 µm).

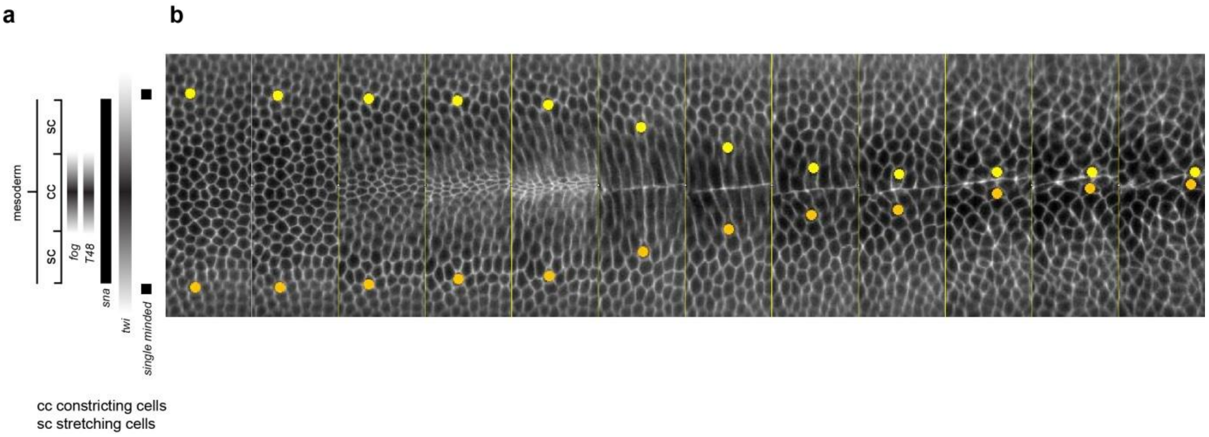

Supplementary Figure 5 │ Gene expression and delimitation of the mesoderm. **a**, Schematic of gene expression patterns in the region occupied by the mesoderm. The expression domain of the gene *snail* precisely defines the lateral edges of the mesoderm. The single cell row adjacent to the mesoderm expresses the gene *singleminded*. The expression domain of the gene *twist*, which encodes the upstream transcription factor necessary for mesoderm invagination, extends slightly beyond the limit of the mesoderm in a graded fashion. The genes *fog* and *T48* are expressed in a graded fashion within the central region of the mesoderm, where their protein products are necessary to trigger apical constriction. **b**, Manual backtracking of the cells that meet at the midline after the mesoderm has invaginated, i.e. the mesectodermal cells that express *singleminded*. Orange and yellow dots represent single mesectodermal cells on the right and left side of the embryo.

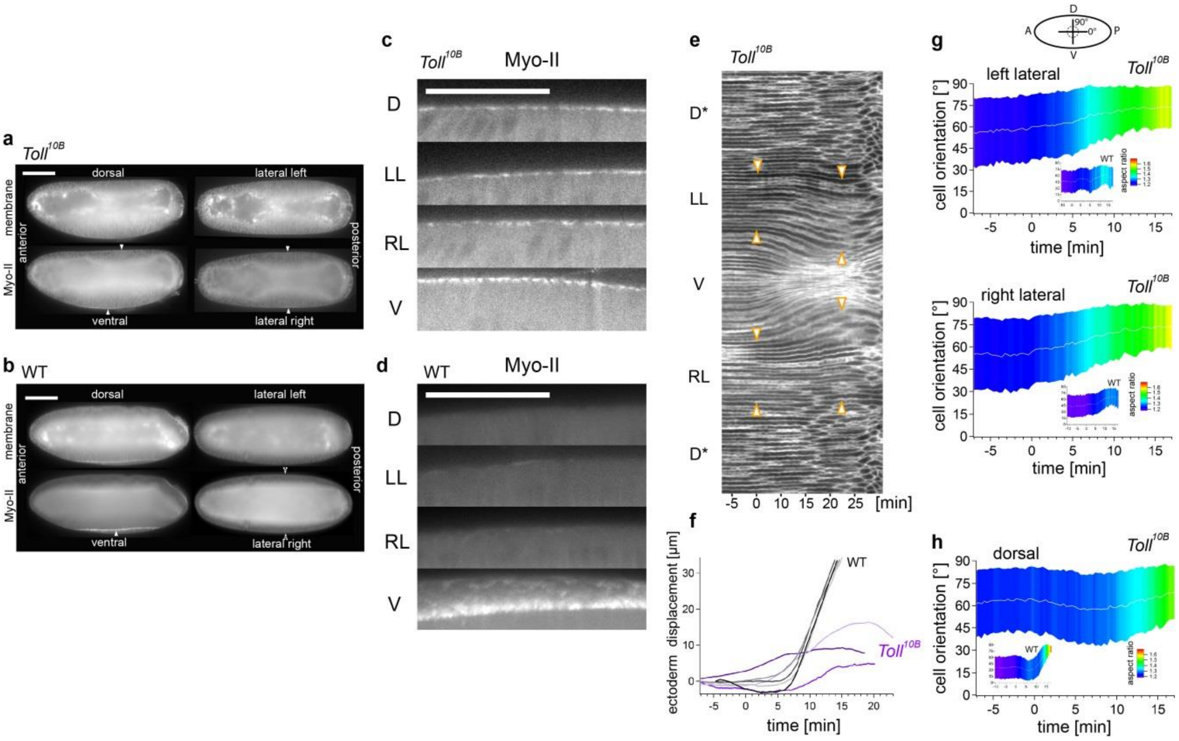

Supplementary Figure 6 │ Cell behaviours in embryos with defective axial patterning systems. a, Sagittal and coronal views of a gastrulating embryo derived from a Toll^{10B} /+ mother, labeled for markers for membrane (top) and Myo-II (bottom). White arrow-heads point to enrichment of Myo-II. **b**, Same as a, but for a wild type embryo. **c**, Sagittal views at D, LL, RL and V positions in an embryo derived from a *Toll*^{10B}/+ mother, labelled for Myo-II. **d**, Same as c**,** but for a wildtype embryo**.** The strongest signal is seen at the apical end of the furrow cells, which at this point have already moved towards the interior. **e**, Kymograph taken at the medial region of a *Toll*^{10B} embryo. Orange arrowheads: positions corresponding to those marked in the wild type embryo in Fig.1. **f**, Lateral cell movement in *Toll*^{10B} (violet) and wildtype embryos (grey). **g**, Orientation of the major axes of the LL and RL (**g**) and dorsal (**h**) cells in a *Toll*^{10B} embryo. The small insets show the data for wildtype embryos for comparison. Colour indicates cell eccentricity. Scale bars, 100 µm.

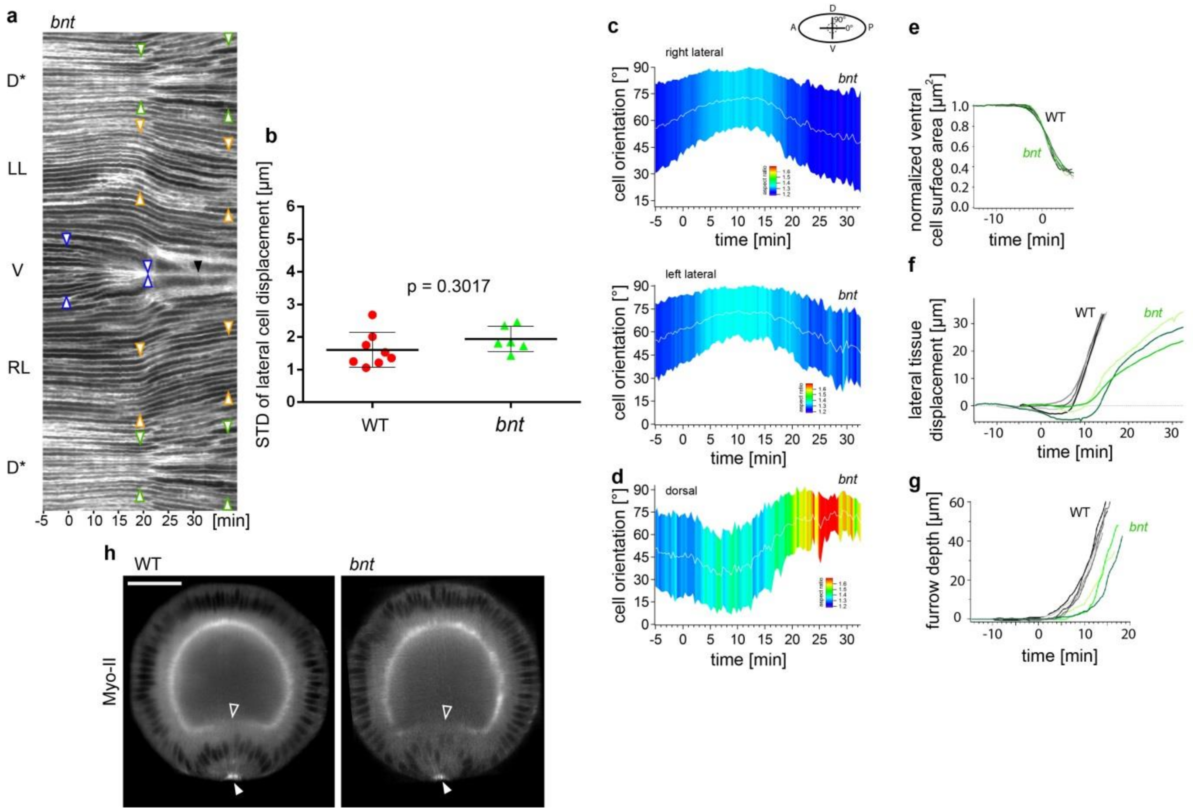

Supplementary Figure 7 │ *bnt* mutant embryo analysis. a, Kymograph of a *bnt* embryo; the spreading dorsal cell population is marked by green arrowheads, the LL and the LR cell populations with orange arrowheads, the constricting ventral cells with blue arrowheads. The black arrowhead points to the ventral midline. **b**, Standard deviation of cell displacement along the DV axis for 6 groups of lateral cells in 3 *bnt* embryos, compared to the data for wildtype embryos as shown in Extended Data Fig. 4b. Measurements and representation as in Extended Data Fig. 4b. Average and standard deviation of major axis orientation for the LR and LL (**c**) and dorsal (**d**) cells. Colour indicates cell eccentricity (red indicates higher eccentricity). **e**, Normalized apical surface area of ventral cells as a function of time. **f**, Lateral cell displacement as a function of time. **g**, Furrow depth as a function of time. In all panels the curves show wild type in black and *bnt* embryos in green. **h**, Cross-section of a wild type and *bnt* embryo during apical constriction of the ventral cells with fluorescently labelled Myo-II. White fill arrows are pointing to similar recruitment of Myo-II and empty arrows to similar depletions of Myo-II. Scale bar 100 µm.

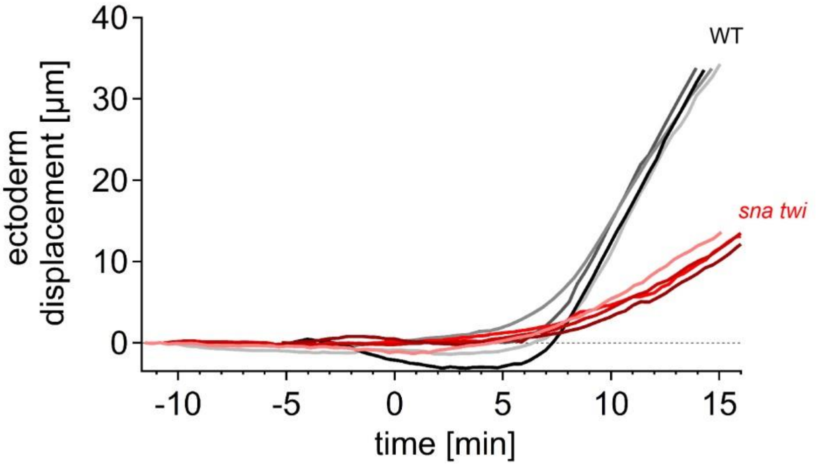

Supplementary Figure 8 │ Displacement of the lateral cell populations in *sna twi* mutant embryos. Displacement of the lateral tissue along the dorsal-ventral axis of the embryo in wild type (black) and *sna twi* mutant (red) embryos as a function of time.

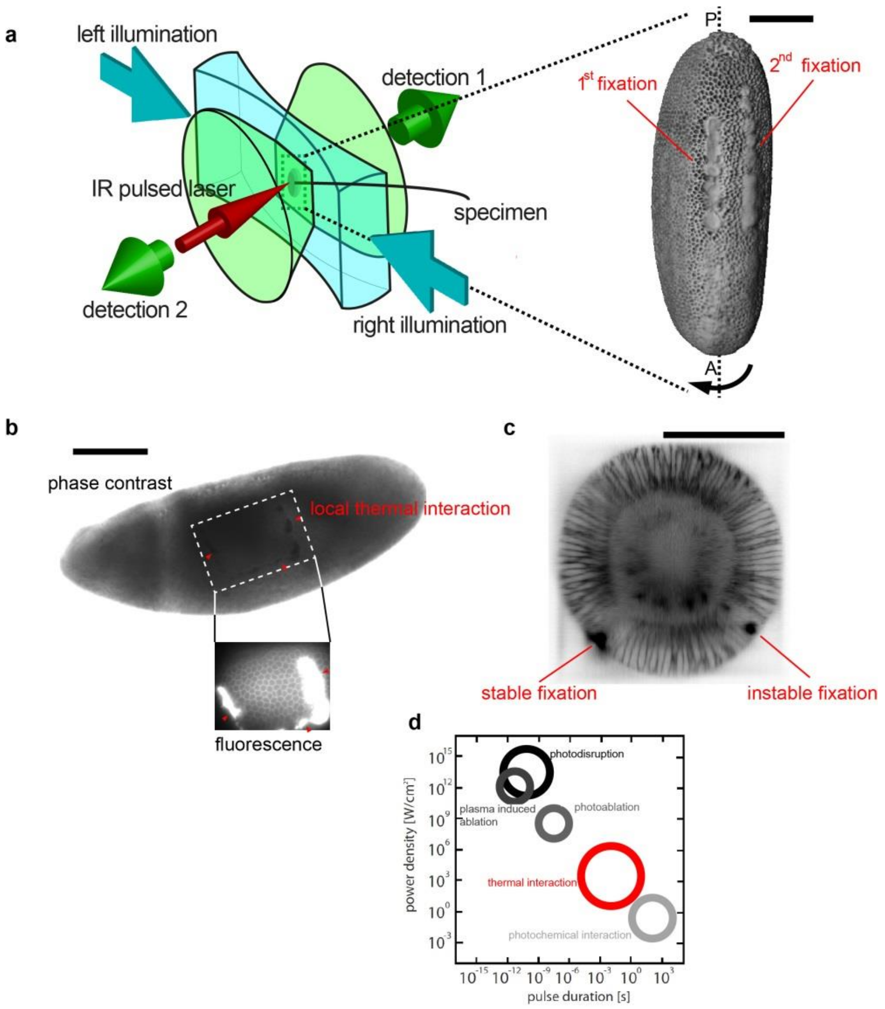

Supplementary Figure 9 │ Infra-red laser-based local thermal interactions result in tissue immobilization. a, Multi-View Selective-Plane-Illumination-Microscopy (MuVi-SPIM) sample chamber in which the two excitation and the two detection objectives are focused. Simultaneous light sheet excitation from both sides is used to double the speed of acquisition. The IR pulsed laser for cell immobilization is injected in the back aperture of one of the two detection objectives and focused on the embryo. Multiple manipulations were performed in series at different sites on the embryo by rotating the sample with a motorized stage. **b**, Phase contrast image of an embryo on which an IR femtosecond laser had been shone along a ‘U’ shaped trajectory (red arrow heads). The inset shows the same embryo imaged with fluorescence microscopy. **c**, Cross-section view of an embryo which had been exposed on two sides to the IR pulsed laser. The fixation on the left is closer to the surface and immobilized the cells permanently. The fixation on the right is less superficial and immobilizes cells only transiently (see Fig. 4f-i). **d**, Map of the

different processes that a laser can cause when interacting with a tissue (drawn after ref. 1). Scale bar 100 µm.

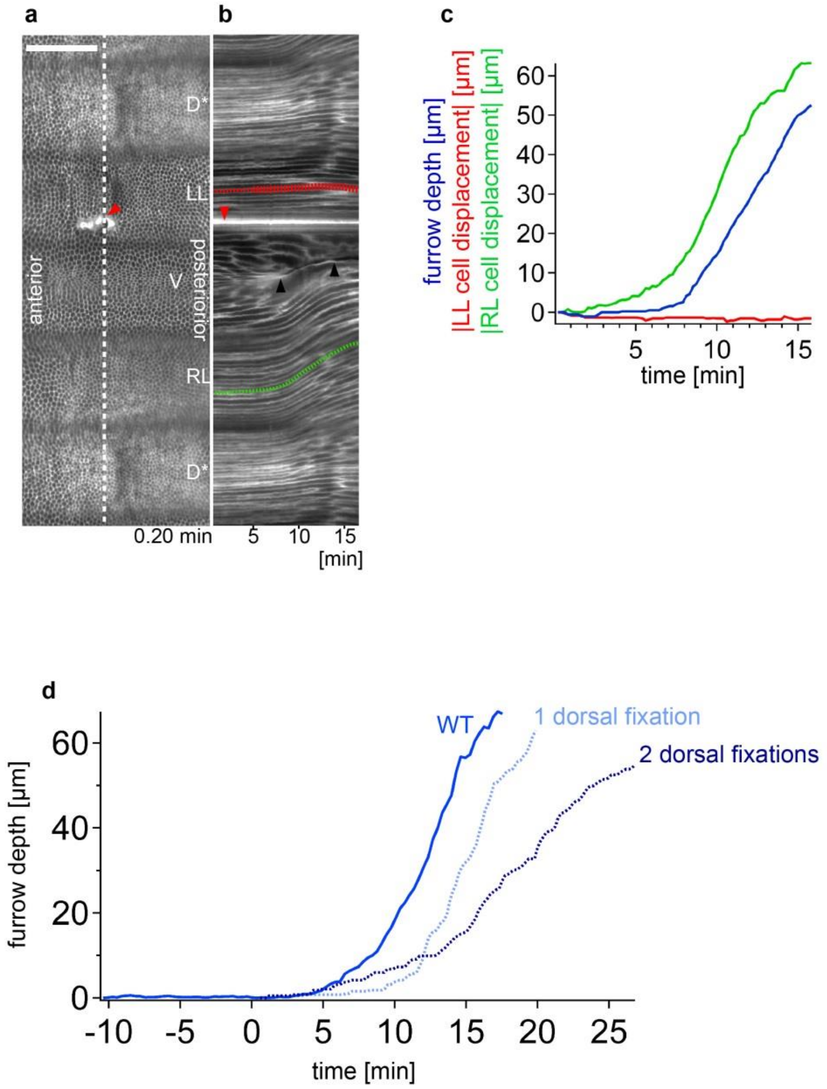

Supplementary Figure 10 │Generating systematic asymmetries by exploiting IR laser-based cell fixation. a, Cylindrical projection of an embryo with laser-induced tissue fixation on the right ventro-lateral side (red arrowhead). **b**, Kymograph taken along the white dashed line in a. The red and green dashed lines highlight lateral cell trajectories on the right and left sides of the embryo. The red line is marked thin during the time in which lateral cells are stationary and thick during the period when lateral cells move in wild type embryos. Black arrowheads indicate the ventral midline position. **c**, Plot of the furrow depth, the absolute value of the RL and LL displacement as a function of time for the embryo shown in b. Scale bar, 100 µm. **d**, Furrow depth as a function of time for control embryos and for embryos on which IR laser-based tissue fixation was performed on the dorsal side (see Fig. 5).

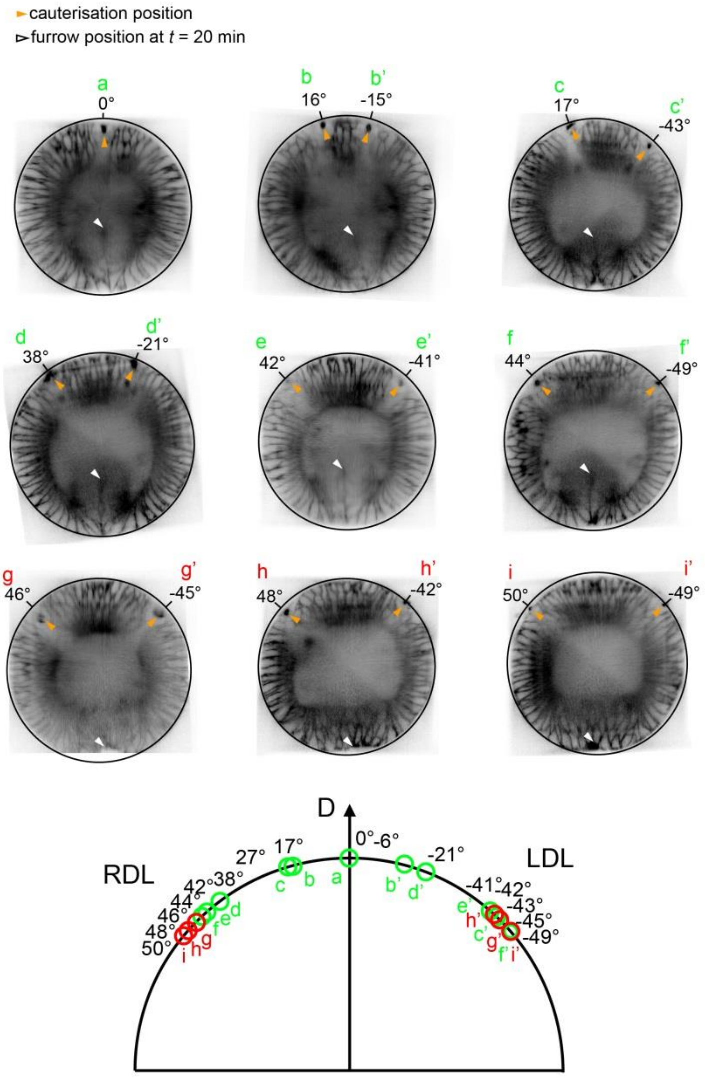

Supplementary Figure 11 │Results of dorsal and dorso-lateral cauterisations. Overview of embryos after dorsal or bilateral cauterisations. The cross-sections are extractions from 3D datasets at t = 20. The letters in the diagram refer to the individual embryos shown above. Embryos a and b are the same as shown in Fig. 5e,f and 5c,d, respectively. Green and red marks cauterisations that do or do not allow furrow formation.

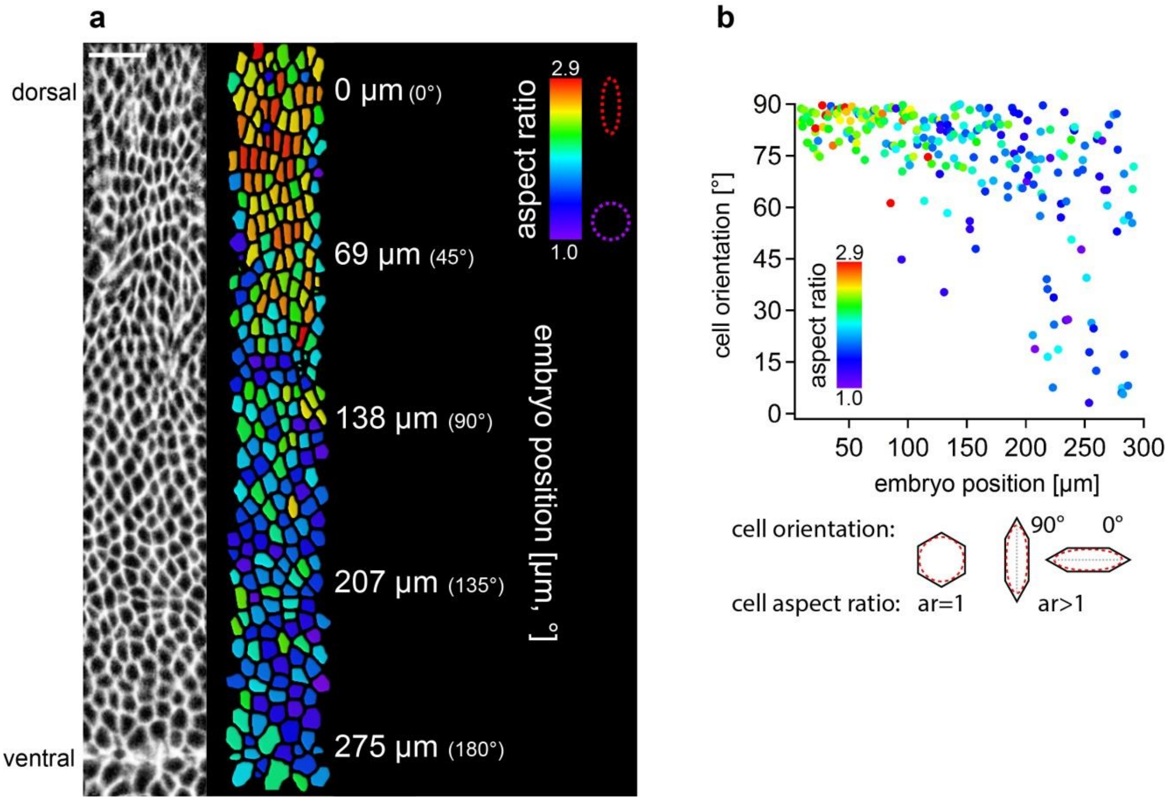

Supplementary Fig 12│Dorsal and ectodermal cell shapes after the completion of mesoderm invagination a. Surface view of an embryo at t = 20 min (left) and segmented cells with aspect ratio plotted in colour (right). Positions around the circumference are measured from the dorsal midline. Note that the angular positions refer to the circumference of the embryo, irrespective of cell movement; the cells at each position were originally positioned at different angles, closer to the dorsal midline. Scale bar: 30 µm **b**. Aspect ratio (colour) and orientation (y axis) of each cell in panel **a.** plotted against the cell's distance from the dorsal midline.

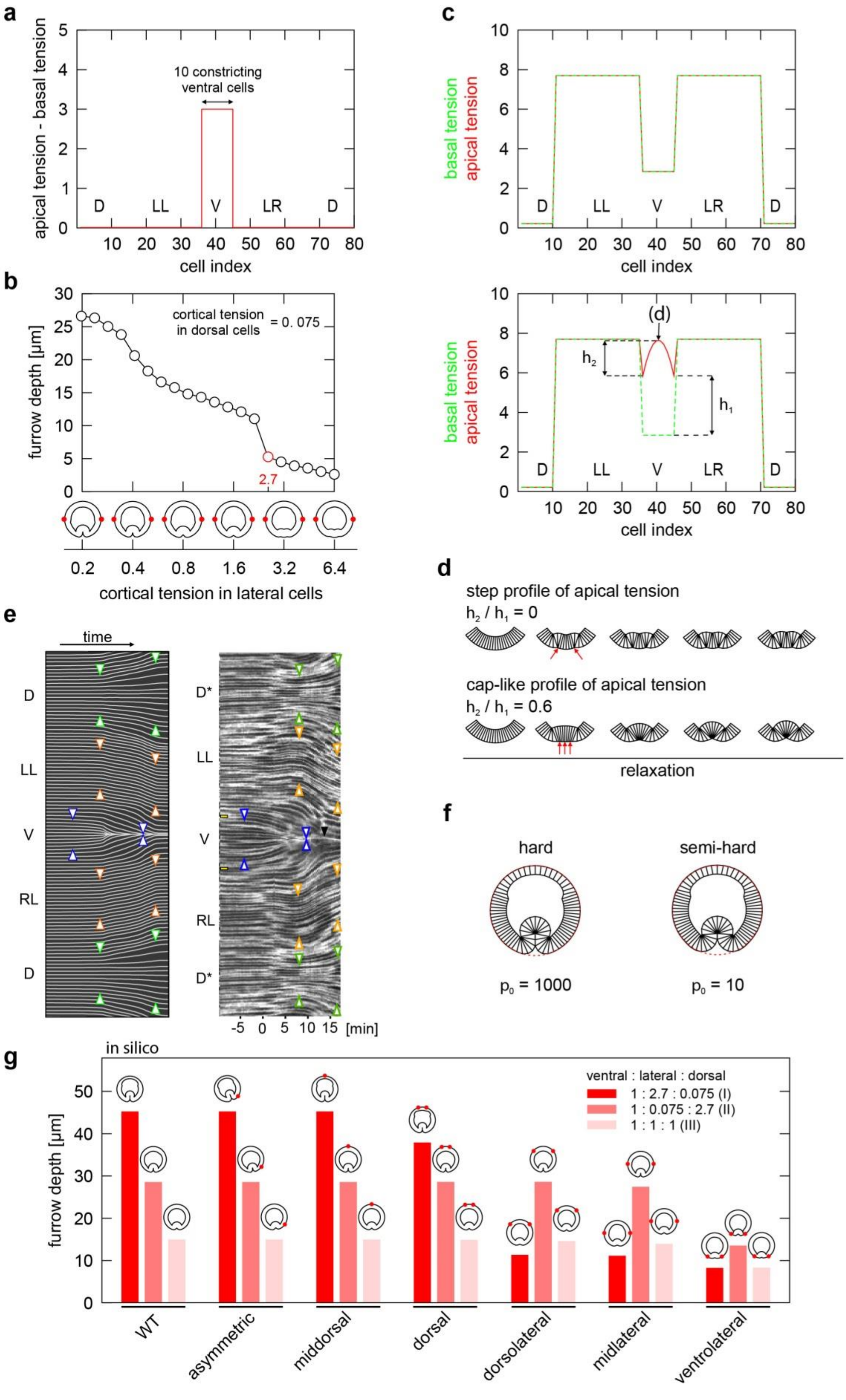

Supplementary Figure 13 │Modelling cell displacement and deformation patterns. The starting profile of differential tension around the circumference of the

model embryo. The circumference is shown along the *x*-axis with the dorsal point at the right and left endpoint of the plot, and the midventral point in the centre. An increase in apical tension imposed on 10 ventral cells generates apical constriction in these cells. **b**, Final shapes of embryos with cauterisations simulated in the midlateral region. The cortical tension in dorsal cells was 0.075, and the cortical tension in lateral cells was varied. For large cortical tensions in lateral cells the ventral furrow cannot fully internalize. **c**, Initial (top) and final (bottom) profile of apical and basal tensions constructed by modelling the experimental results as reported in the main text. **d**, A step-like apical tension profile does not give rise to a homogeneous constriction of ventral cells observed experimentally (top) but a cap-like profile (bottom) does. **e**, Comparison of theoretical (left) and experimental (right) kymographs. Green, orange, and blue arrows point to trajectories of pairs of cells in the dorsal, lateral, and ventral tissue, respectively. **f**, Final shapes of the embryo with a hard and a semi-hard vitelline membrane (p_0 = 1000 and 10, respectively). **g**, Furrow depth for different *in silico* cauterization experiments. The experimental results are correctly reproduced by our model using parameter set I but not by the parameter sets II and III.

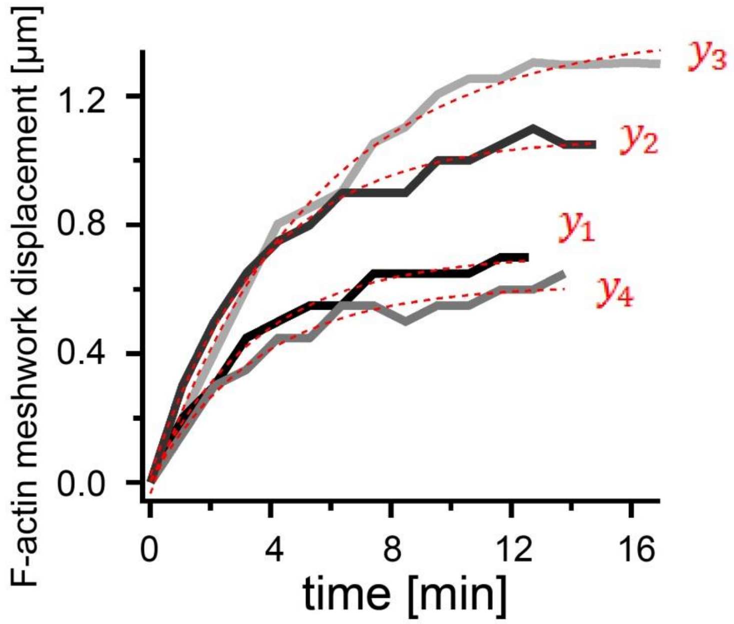

fitting function to determine the decay coefficient, invTau

$$y = y_0 + A\exp(-invTau \cdot x)$$

values of invTau for ventral tissue in 4 embryos

$$invTau_1 = 0.28255 \pm 0.0235$$
$$invTau_2 = 0.27986 \pm 0.035$$
$$invTau_3 = 0.18714 \pm 0.0118$$
$$invTau_4 = 0.27084 \pm 0.0191$$

Supplementary Figure 14 │ Defining the decay coefficient for the recoil of ventral tissue. Recoil curves of the ablated ventral tissue fitted with an exponential function (red line) to define the decay coefficient.

Supplementary Reference

1. Niemz, M.H. *Laser-Tissue Interactions*, (Springer, 2004).
